# Supplementary material for: Worldwide genetic variation of the IGHV and TRBV immune receptor gene families in humans
Source: Life Sci Alliance. 2019 Feb 26;2(2):e201800221. doi: 10.26508/lsa.201800221 (PMC6391684; doi:10.26508/lsa.201800221)
Supplement: Supplementary file 1 [file LSA-2018-00221_Supplementary_Text.pdf]

Supplementary Text

Worldwide genetic variation of the IGHV and TRBV immune receptor gene families in humans

Shishi Luo[1,2,*], Jane A. Yu[1,*], Heng Li[3], Yun S. Song[1,2,4,†]

[1]Computer Science Division, University of California, Berkeley, Berkeley, CA 94720, USA
[2]Department of Statistics, University of California, Berkeley, Berkeley, CA 94720, USA
[3]Broad Institute of Harvard and MIT, Cambridge, MA 02142, USA
[4]Chan Zuckerberg Biohub, San Francisco, CA 94158, USA

[*]These authors contributed equally to this work

[†]To whom correspondence should be addressed: yss@berkeley.edu

Read Mapping

For each individual's whole genome sequencing FASTQ file, we retained reads from two separate procedures: (i) reads that map to the IGHV and TRBV loci on the GRCh37 reference, (ii) reads that map to a list of functional IGHV and TRBV (from the online IMGT database).

Procedure (i) used bwa mem (https://github.com/lh3/bwa) with default parameters, e.g.:

```
bwa mem grch37.fa read1.fq read2.fq | gzip -3 > aln-pe.sam.gz
```

Procedure (ii) used minimap (https://github.com/lh3/minimap):

```
minimap -w1 -f1e-9 imgt_ighv.fa.gz read-se.fa.gz > out_ighv.mini
minimap -w1 -f1e-9 imgt_trbv.fa.gz read-se.fa.gz > out_trbv.mini
```

Although procedure (i) was sufficient in our previous study (Luo, Yu, and Song 2016) for identifying all the IGHV genes in an individual, it was clear that for the Simons dataset, the mapped reads were biased to the GRCh37 reference (Fig. S12). This is possibly due to differences in mapping algorithms in the different sample sets. For this reason, procedure (ii) was needed instead to 'catch' the reads that are not in the reference genome but which map to known IGHV and TRBV gene segments. Such gene segments for the GRCh37 reference genome are IGHV7-4-1, IGHV4-4, IGHV5-10-1, IGHV4-30-2, IGHV4-30-4, IGHV4-38-2, IGHV1-69-2, IGHV3-NL1, TBRV5-8, TRBV6-2, TRBV7-2, TRBV7-7, TRBV7-9, TRBV10-3, TRBV11-3, TRBV12-3, TRBV12-5, TRBV13, TRBV14, TRBV15, TRBV16, and TRBV18.

Thus, unless otherwise stated, all our results are based on reads that were mapped using procedure (ii) *only*.

Read filtering

IGHV

We will call the set of reads obtained via procedure (ii), R_{IMGT}. R_{IMGT} includes reads from all parts of an individual's genome (functional, pseudo, and orphon genes) that share some sequence similarity with the list of functional IGHV alleles (Giudicelli, Chaume, and Lefranc 2005). Our goal is to remove reads from pseudogenes and orphon genes and also resolve instances where a single read maps equally well to more than one functional gene. We devised gene-specific filtering rules to minimize erroneously mapped reads.

Operationally indistinguishable IGHV genes
First we established that some IGHV genes are operationally indistinguishable from each other. Specifically, IGHV genes at distinct genomic locations have alleles that are highly similar (more than 95% nucleotide similarity). These indistinguishable sets are:

- {IGHV3-23, IGHV3-23D}
- {IGHV3-30, IGHV3-30-3, IGHV3-30-5, IGHV3-33}
- {IGHV3-43, IGHV3-43D}
- {IGHV3-53, IGHV3-66}
- {IGHV3-64, IGVH3-64D}
- {IGHV1-69, IGHV1-69D}
- {IGHV2-70, IGHV2-70D}

The basis for grouping these IGHV genes together was detailed in (Luo, Yu, and Song 2016). Note however, that here we do not use the operational clusters that mix IMGT segment labels so as to minimize confusion with IMGT nomenclature. For the purposes of our study, we do not attempt to discriminate between IGHV genes in the above sets.

Taking the full set of 54 IMGT functional gene segments and combining those in the above sets gives a set of 45 operationally distinguishable functional IGHV segments.

Discarding reads that map uniquely to pseudogenes and orphon genes
Our filtering begins by performing IgBLAST on all these reads against an expanded set of IGHV alleles that includes orphon genes and pseudogenes. Once we obtain the results of the IgBLAST procedure, we first discard all reads for which all the top hits are alleles of a single orphon gene or pseudogene.

For example, consider the following read:

```
>HS2000-1266_146:7:1206:16772:59318/1
GCTTGAGTGGATGGGATGGATCAACACTTACAATGGTAACACAAACTACCCACAGAAGCT
CCAGGGCAGAGTCACCATGACCAGAGACACATCCACGAGC
```

This read matches the orphon gene IGHV1/OR15-2*01 exactly, and is therefore likely to have come from the orphon gene. However, it was originally included in R_{IMGT} because it is similar to positions 132-191 of functional allele IGHV1-18*01, deviating by four nucleotides. Having established through IgBLAST that there is little ambiguity about where this read comes from, we discard it.

Functional IGHV genes to which 100 bp reads map uniquely
The set of reads we have left, call it R_{IMGT_fcn}, consist of reads that either uniquely map to a functional IGHV gene, or map equally well to regions of functional and pseudogenes/orphon genes. The former category is most straightforward to deal with. The 16 (out of 45) operationally distinguishable functional IGHV genes for which the reads in R_{IMGT_fcn} can be unambiguously mapped are:

IGHV6-1, IGHV3-9, IGHV5-10-1, IGHV1-18, IGHV3-20, IGHV1-24, IGHV2-26, {IGHV3-43, IGHV3-43D}, IGHV1-45, IGHV3-49*, IGHV5-51, IGHV1-58, {IGHV1-69, IGHV1-69D}, IGHV1-69-2*, IGHV3-72, IGHV3-73.
*These gene segments have inflated coverage in a subregion, see Supplementary Information Figure 1.

Functional IGHV genes to which 100 bp reads are not uniquely mapped
For reads that map equally well to more than one IGHV gene (functional or otherwise), we do not have enough information to assign reads to genes with 100% confidence. If we assign a read to all the functional genes that

are tied for top hit, some functional genes will have extra reads assigned to them. If we only assign the reads with a unique top hit, then we will lose information on functional genes that share subsequences of substantial length with other genes. Compounding matters is the fact that the presence/absence and copy number of IGHV genes can vary from individual to individual, so that an approach that works in one individual may have a different effect in another.

To determine the rules for filtering out reads that would have the least error on average, we used the read coverage profile for each IGHV segment, aggregated over all individuals in our sample. We compare the coverage profiles for a given segment under two read filtering rules:
(A) ‘All top hits’: keep all reads which have that segment as a top hit (unique or tied), or
(B) ‘Unique top hits’: keep only reads which have that segment as a unique top hit.

As an example of a well-behaved case, here is the profile for IGHV6-1:

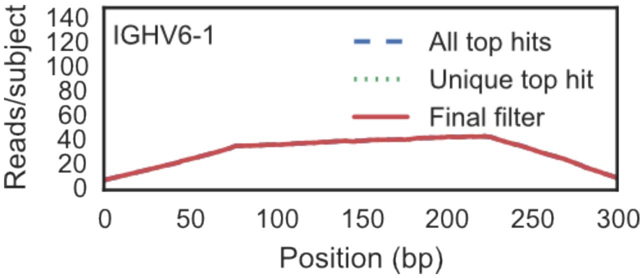

Note that the per-base read coverage, averaged over all 109 individuals (from blood/saliva samples), matches very closely with what is expected theoretically. Specifically, the coverage decreases linearly towards the edges because reads that only partially cover the segment will have lower mapping scores and therefore were not in our original set R_{IMGT}. The per-base read coverage of around 40 is also consistent with the median genomic coverage of 42 across the full Simons sample (Supplementary Data Table 1 of (Mallick, Li, Reich, et al. 2016)). This profile is evidence the set of reads that map to IGHV6-1 does not contain reads from other similar IGHV genes, which is consistent with our earlier observation that IGHV6-1 is a gene to which reads map uniquely.

Supplementary Information Figure 1 contains the profiles of all the operationally distinguishable gene segments. We will describe a few representative examples here.

Example: IGHV1-2
Some of the reads that map to IGHV1-2 also map equally well (in lengths >75 base pairs) with alleles of IGHV1-8 and IGHV1-OR15-1 (an orphon gene on chromosome 15). Indeed, the per-base read coverage of IGHV1-2 average across our sample of 109 individuals shows that there is an overabundance of reads mapping to the first 100 base pairs of the gene. However, when we only keep the reads for which IGHV1-2 is the unique top hit, the per-base read coverage more closely resembles what is expected theoretically.

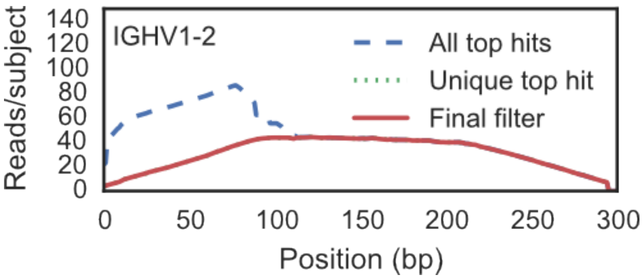

That this correction works suggests that reads mapping equally well to IGHV1-2 and another gene in fact do not align with either very well, and therefore should be discarded as most likely not coming from IGHV1-2.

Similar reasoning holds for
IGHV1-3, IGHV7-4-1, IGHV2-5, IGHV3-7, IGHV1-8, IGHV3-11, IGHV3-13, IGHV3-21, IGHV3-23, IGHV4-30-2, IGHV4-30-4, IGHV3-30, IGHV4-34, IGHV1-46, IGHV3-48, IGHV3-53, IGHV3-64, IGHV2-70, IGHV3-NL1

It should be noted that we achieve varying levels of success. In particular for IGHV3-15, there was still quite elevated coverage after performing this step (see Supplementary Information Figure 1). In other cases, we probably undercount the reads for some of these genes, giving us a conservative estimate of the segment copy number.

Example: IGHV4-31
It was clear from the per-base coverage of IGHV4-31 that if we discarded all the reads that mapped equally well to other genes, we would not obtain full sequence reconstruction of the gene even for the individuals it is present in. Moreover, counting all the reads that map to IGHV4-31 did not suggest we were over counting. Thus, for IGHV4-31, we kept all the reads that had IGHV4-31 as a top hit.

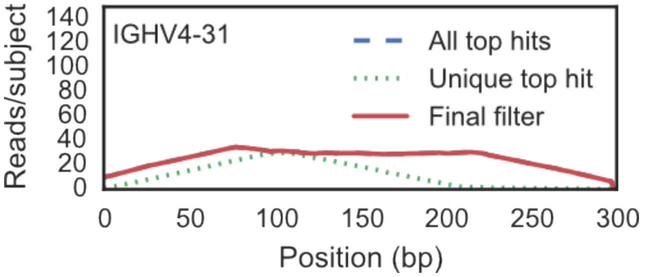

This reasoning also held for IGHV4-39 and IGHV4-28.

Example: IGHV3-74
Among all the functional IGHV segments, IGHV3-74 is unique in that it shares a long subsequence with an orphon gene that seems to be present in single copy on all haplotypes. Specifically, positions 46-182 (137 base pairs) of IGHV3-74 (alleles *01 and *02, the two most common alleles) are identical to IGHV1-OR/16-13*01. In this special case, we can toss a fair coin to determine whether to assign a read to IGHV3-74 and be fairly confident we are counting accurately.

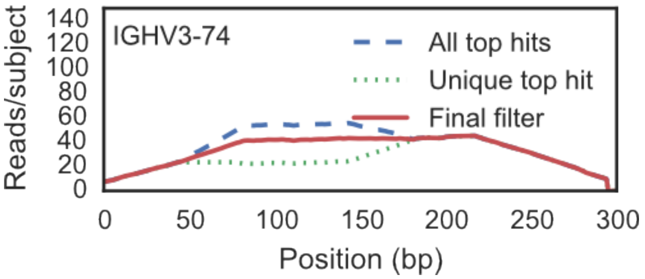

Example: IGHV4-4, IGHV4-59, IGHV4-61
For IGHV4-4, IGHV4-59, IGHV4-61, and to a lessor extent IGHV4-38-2, there are too many subregions that exactly match other IGHV genes. Counting only reads with single top hit is a severe underestimate, and counting reads with ties for top hits is a severe overestimate. Thus, for lack of a better option, we sample the IGHV gene from the top hits at random, in proportion to the coverage of the top hits in the region outside the mapping region. Unlike the case with IGHV3-74, we know that the copy number of the other genes that share subsequences will differ between individuals. Hence, we know this approach is flawed. However, it gives our

best estimate for the reads that come from these IGHV genes and our overall conclusions are not sensitive to the calls we make for these genes.

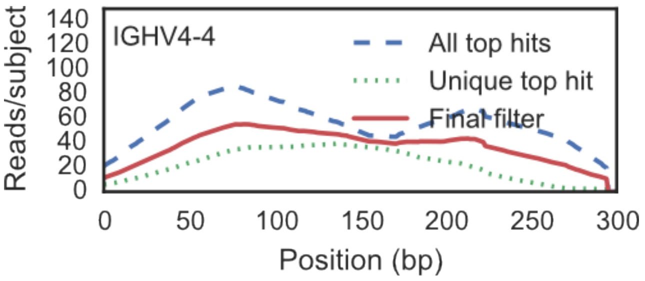

After the above filtering steps have been performed, for each operationally distinguishable IGHV gene indexed by i, we have a set of reads, call it $R_{filtered,i}$,

TRBV

There were fewer issues in read filtering for the TRBV locus compared to the IGHV locus.

Operationally indistinguishable TRBV genes

As with the IGHV locus, there are some TRBV genes that are operationally indistinguishable from each other when using 100 bp reads. These are:

- {TRBV4-2, TRBV4-3}
- {TRBV6-2, TRBV6-3}
- {TRBV12-3, TRBV12-4}

For our purposes, we do not attempt to distinguish between segments within these sets.

Taking the full set of 48 IMGT functional TRBV segments and combining those that are in the above sets gives 45 operationally distinguishable functional TRBV segments. Coincidentally, this is the same number of operationally distinguishable functional IGHV segments.

Discarding reads that map uniquely to pseudogenes and orphon genes

As with the IGHV reads, we begin by performing IgBLAST (for T cell receptors) on all the reads against an expanded set of TRBV alleles that includes orphon genes and pseudogenes. Once we obtain the results of the IgBLAST procedure, we first discard all reads for which the top hits are all alleles of a single orphon gene or pseudogene.

For example, consider the following read:

```
>HS2000-1266_146:7:1205:14671:84576/2
GCTCCGGGCTTAGTGCTGTCGTCTCTCAACATCCGAGCAGGGTTATCTGTAAGAGTGGAA
CCTCTGTGAACATCGAGTGCCGTTCCCTGGACTTTCAGGC
```

This read matches the orphon gene TRBV20/OR9-2*01 exactly, and is therefore likely to have come from the orphon gene. However, it was originally included in R_{IMGT} because it matches positions 1-89 of functional allele TRBV20-1*02, deviating by two nucleotides. Having established through IgBLAST that there is little ambiguity about where this read comes from, we discard it.

Functional TRBV genes to which 100 bp reads map uniquely

The set of reads we have left, call it R_{IMGT_fcn}, consist of reads that either uniquely map to a functional IGHV gene, or map equally well to regions of functional and pseudogenes/orphon genes. The former category is most

straightforward to deal with. The 38 (out of 45) operationally distinguishable functional TRBV genes for which the reads in R_{IMGT_fcn} can be unambiguously mapped are:

TRBV2, TRBV3-1, TRBV4-1, {TRBV4-2, TRBV4-3}, TRBV5-1, TRBV5-4, TRBV5-5, TRBV5-8, {TRBV6-2, TRBV6-3}, TRBV6-4, TRBV6-6, TRBV7-2, TRBV7-3, TRBV7-4, TRBV7-6, TRBV7-7, TRBV7-8, TRBV7-9, TRBV9, TRBV10-1, TRBV10-2, TRBV10-3, TRBV11-1, TRBV11-2, TRBV11-3, {TRBV12-3, TRBV12-4}, TRBV12-5, TRBV13, TRBV14, TRBV15, TRBV16, TRBV18, TRBV19, TRBV20-1, TRBV27, TRBV28, TRBV29-1, TRBV30

Functional TRBV genes to which 100 bp reads are not uniquely mapped
As with the IGHV genes, we also have TRBV genes for which we cannot determine the correct reads with 100% confidence. This is a much smaller set of genes and again we compare the coverage profiles for a given TRBV gene under two read filtering rules:
(A) 'All top hits': keep all reads which have that segment as a top hit (unique or tied), or
(B) 'Unique top hits': keep only reads which have that segment as a unique top hit.

Supplementary Information Figure 2 contains the profiles of all the TRBV genes with summaries of how we cleaned up the reads mapping to them. We will describe two representative examples here.

Example: TRBV5-5
As with IGHV3-74, taking all top hits versus taking only unique top hits gave coverage profiles that were equally displaced above and below the theoretical expectation. Note that the displacement is much smaller than for IGHV3-74. Thus, for each read that has multiple top hits including TRBV5-5, we sample uniformly over the segments that are the top hits.

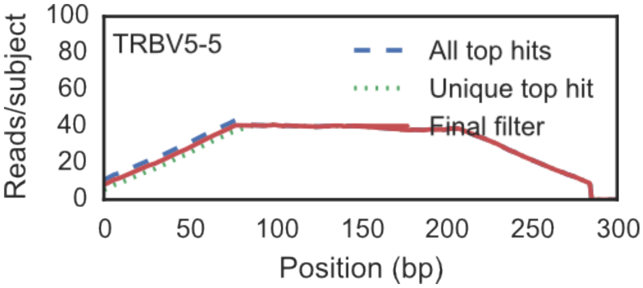

This strategy was also used for TRBV6-1, TRBV6-5, TRBV6-8, TRBV6-9.

Example: TRBV24-1
This segment had a highly unusual coverage profile. For a lack of a better strategy, we also sampled uniformly over the segments that are top hits for reads with multiple top hits.

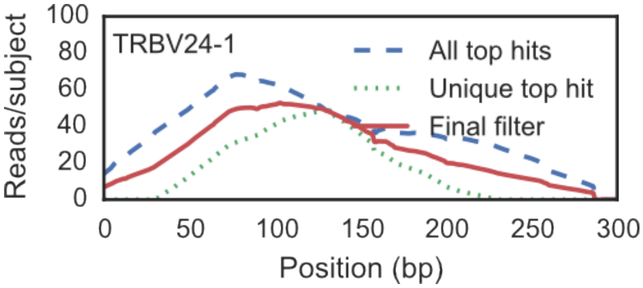

Example: TRBV25-1

This segment also had a strange profile, but for a different reason. As with IGHV3-15, there seems elevated coverage, perhaps due to be as yet uncharacterized segments that share sequence similarity (see Supplementary Information Figure 2).

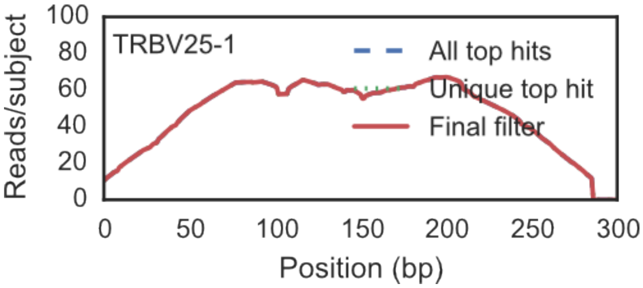

After the above filtering steps have been performed, for each operationally distinguishable TRBV gene indexed by i, we have a set of reads, call it $R_{filtered,i}$,

Copy number from kmer coverage

Suppose the kmer coverage of the reconstructed contig for a gene segment in an individual is *d* and the genome-wide coverage is *g*. Our point estimate for copy number would be:

$$c = d \times \frac{5}{4} \times \frac{4}{3} \times \frac{1}{g} \times 2$$

where 5/4 is the factor to convert 21-mer coverage of 100 bp to per-base coverage, 4/3 corrects for the fact that the trapezoidal coverage profiles (see Supplementary Information Figures 1 and 2) gives per-base coverage depth that is ¾ of true uniform coverage depth, $1/g$ normalizes by the genome-wide average coverage depth, and we multiply by 2 so that a *c* of 2 corresponds to one copy per haplotype (two copies per individual).

However, we found that in practice, the distributions for copy number using this formula consistently led to underestimates of copy number across the segments. For example the bulk of our point estimates for well-behaved and typically single copy per haplotype genes such as IGHV6-1, IGHV2-5, and IGHV5-51, were clustered just below the value two. The likely explanation is that the genome-wide coverage value we used is an overestimate of the true read coverage depth. We found that by multiplying the genome-wide coverage by 0.9, we obtained distributions for copy number that clustered more symmetrically around integer values, i.e., our point estimates were calculated as:

$$c = d \times \frac{5}{4} \times \frac{4}{3} \times \frac{1}{0.9g} \times 2$$

Finally, to obtain an integer from this point estimate, we simply round to the nearest whole number. The exception to this is when the point estimates look systematically biased across all the individuals, as is the case with IGHV7-4-1, IGHV3-7, IGHV3-49, and IGHV1-69-2. In these three cases, we calculate the mean shift of the points from integer values and move them all down by that shift before rounding to the nearest integer.

Hierarchical clustering (for Figures 2 and 3)

To call the copy number variants for common polymorphisms involving multiple genes, we use the point estimates for copy number. The reason we do not round the point estimates to whole integers is that the rounding process may introduce additional error into our variant calls. In order to leverage knowledge of existing multi-gene CNVs to improve our copy number estimates, we performed a hierarchical clustering (scipy.cluster.hierarchy) on the set of individuals, representing each individual as a vector comprised of the

copy number estimate for each gene in the polymorphisms in Figure 2 and 3. For example, a scaled coverage value of 2.5 for IGHV1-69 could be consistent with a copy number of 2 or 3, but if IGHV2-70 has scaled coverage value of 2.1, it is more likely that IGHV1-69 is copy number 2. Furthermore, given that IGH1-69-2 had no reads mapping to it, we would be even more certain. Note that we did not use this method for any CNVs beyond those in Figure 2 and 3 involving two or more operationally distinguishable gene segments, because it would merely bin the copy number calls by intervals (e.g. Fig. S3B, Fig. S5B, Fig. S5C).

Determination of two-copy segments

To determine the set of 11 two-copy IGHV genes and 40 two-copy TRBV, we selected genes which are two copies in the vast majority of individuals in our sample and for which there is minimal read-mapping ambiguity. In other words, we selected genes for which the "Notes" column in Supplementary Information Figure 1 and Supplementary Information Figure 2 have "No subsequences shared with other known IGHV genes" and "Predominantly single copy per haplotype".

Exceptions to this rule include IGHV3-74, TRBV5-5, TRBV5-8, TRBV6-1, TRBV6-5, TRBV6-8, and TRBV6-9. For these segments, the red solid line (the read profile for the filtered set of reads in Supplementary Information Figure 1 and Supplementary Information Figure 2) lies roughly halfway between the green dotted line (the read profile resulting from taking the unique top hit) and the blue dashed line (the read profile resulting from taking all top hits). In such cases, the red line results from tossing a fair coin to determine which of the two genes to assign a given read. Because the resulting red read profile aligns with the expected trapezoidal shape, we believe that the alternative gene which shares the subsequence is present at the same copy number. Additionally, since the shared subsequence is identical in both genes, this should not lead to significant mis-mapping errors. TRBV7-4 and TRBV7-6 are also included in the set of two-copy genes since these two genes share a subsequence that is not 100% identical, and because the blue and green lines align, implying that the shared subsequence does not lead to mis-mapping. Consequently, we have included these operationally distinguishable gene segments in the set of two-copy segments, given that they are also predominantly single-copy per haplotype.

Alternative procedure for unphased variants from HapCUT2

When few or no reads covered two SNPs, HapCUT2 failed to phase the full segment. To account for this issue, we took all combinations of completely phased blocks as potential allele sequences. As a toy example, consider a sequence of length three, each position having an unphased polymorphic site with A on one chromosome and G on the other. Taking combinations results in four pairs of haplotypes (AAA, GGG), (AGA, GAG), and (AAG, GGA), and (AGG, GAA). With the resulting pairs of phased sequences, we compute the probability of observing each candidate pair of allele sequences (a_1, a_2), which is calculated by taking the maximum of $P(a_1|a_2)P(a_2)$ and $P(a_2|a_1)P(a_1)$, where $P(a_x|a_y)$ is the fraction of individuals observed to have both allele sequences a_x and a_y out of all individuals observed to have allele a_y, and $P(a_x)$ is defined as the fraction of observations of a_x out of all individuals. If all candidate pairs of allele sequences were not observed in any other individual, then the following was selected as the individual's phased allele sequence pair: the pair of haplotypes that contains allele sequence a_x, which is the allele with the greatest frequency. If all candidate sequences a_x for an individual were not observed in the rest of the population, then no allele sequence was reported for that segment for that individual. This, however, does not affect our analysis of presence/absence of gene segments.

Novel allele/SNV notation

Alleles were given IMGT names if their sequence exactly matched an allele in the IMGT database. Otherwise, the name of the closest allele was given with an appended suffix for each mutational difference from the closest IMGT allele. Each mutation is represented as {reference base pair}{alternative base pair}{position}{reference amino acid}{alternative amino acid}. For example, allele '*IGHV1-18*01_ag168ND*' denotes an allele whose

sequence is that of IMGT allele *IGHV1-18*01*, but with a 'g' at position 168 rather than an 'a'. The two letters following the position is the amino acid corresponding to the reference base pair 'a' and the amino acid corresponding to the mutation 'g', respectively (the reference amino acid is N and the alternate amino acid is D). If the sequence was equally close to more than one IMGT allele, the IMGT allele of lowest numeric order was chosen. Alleles were called "novel" if it differed in at least one nucleotide from an existing IMGT allele. For SNVs, the notation is similar but mutations are represented simply as {alternative base pair}{position}.

Method performance

The method utilized in this work is an extension of the method used in (Luo, Yu, and Song 2016), which provides tests on simulated data and an application of the method to a sixteen-member pedigree of European descent. To summarize, we simulated reads using all combinations of read length (70, 100, 250bp), reference genomes (GRCh37 and GRCh38), and coverage depth (30x, 40x, 50x), and measured the recall, the fraction of operationally distinguishable gene segments that are correctly called by the pipeline. All except 2 of the 18 combinations demonstrated 100% recall, and the remaining 2 simulations had recall of 97%.

In this work, we have added steps towards haplotype phasing since the method from only constructed a single contig. In addition, we have now also performed simulations for both IGHV and TRBV. Simulating with the set of genes from 109 individuals for IGHV and 286 for TRBV that we empirically inferred from the SGDP dataset, we ran the reads through our pipeline to identify the accuracy rate. Specifically, out of all the alleles identified, we measured how many matched what was originally simulated. For IGHV this was 95.92% and for TRBV this was 98.43%. However, we emphasize again that other approaches may be more appropriate if the goal is to genotype a single individual at base pair resolution, rather than a large set of individuals at coarser resolution.

Analysis of IGHV and TRBV gene segments in 13 vertebrate species

Within-species analysis

For this analysis, we first measured between-segment diversity of the nucleotide sequences annotated in the IMGT human gene table located at http://www.imgt.org/IMGTrepertoire/index.php?section=LocusGenes&repertoire=genetable&species=human&group=IGHV. In this study, we used only those functional alleles for which a position in the locus was recorded. Note that this resulted in a list of one allele sequence per gene segment. As a measure for diversity, we used pairwise global alignment with default BLASTN parameters (match=1, mismatch=–3, gap opening=–5, gap extension=–2). Alignment was done in python using the *pairwise2* module in the Biopython package at biopython.org (Cock, Antao, Chang, Chapman, Cox, Dalke, Friedberg, Hamelryck, Kauff, Wilczynski, Hoon, and L 2009). Averaging over all possible pairs for IGHV gives a mean score of –44 and for TRBV this was –179.

We then expanded our analysis to thirteen vertebrate species, including human. For this analysis, we used amino acid sequences for IGHV segments and TRBV segments obtained from vgenereportoire.org (Olivieri and Gambón-Deza 2014). For each species, the IGHV gene segments and TRBV gene segments were downloaded for one reference genome for that species. For reasons beyond our control, only the amino acid sequences and not the nucleotide sequences were available on the website. Species are: *homo sapiens* (human, ABBA00000000.1), *pan troglodytes* (chimpanzee, AADA00000000.1), *gorilla gorilla gorilla* (gorilla, CABD000000000.3), *pongo abelii* (orangutan, ABGA00000000.1), *macaca mulatta* (rhesus macaque, AANU00000000.1), *mus musculus* (mouse, AAHY00000000.1), *canis lupus familiaris* (dog, AAEX00000000.3), *oryctolagus cuniculus* (rabbit, AAGW00000000.2), *orcinus orca* (orca, ANOL00000000.2), *monodelphis domestica* (opossum, AAFR00000000.3), *ornithorhynchus anatinus* (platypus, AAPN00000000.1), *crocodylus porosus* (crocodile, JRXG00000000.1), *danio rerio* (zebrafish, CABZ00000000.1). The results are displayed in Figure 6 in the main text.

Between-species analysis

Given larger diversity between gene segments in TRBV than IGHV, we measured diversity between gene segments in humans and dogs. The set of nucleotide sequences used for humans were the same as those used previously in the human within-species analysis. The set of nucleotide sequences used for dogs are curated at http://www.imgt.org/IMGTrepertoire/index.php?section=LocusGenes&repertoire=genetable&species=dog&group=IGHV. Again, only functional alleles were utilized in the study. Because identifying orthologous IGHV/TRBV genes in two species is very challenging, for each gene in the human reference, we computed the average alignment score to all other genes in the dog reference. For each gene in the human reference we could have used the alignment score to the closest aligning gene in the dog reference, but this might underestimate the amount of true gene diversity. Taking averages, we computed a mean score of –91 for IGHV and –306 for TRBV.

References

Cock, Peter J. A., Tiago Antao, Jeffrey T. Chang, Brad A. Chapman, Cymon J. Cox, Andrew Dalke, Iddo Friedberg, Thomas Hamelryck, Frank Kauff, Bartek Wilczynski, et al. “Biopython: Freely Available Python Tools for Computational Molecular Biology and Bioinformatics.” *Bioinformatics* 25 (11): 1422–23. doi:10.1093/bioinformatics/btp163.

Giudicelli, Véronique, Denys Chaume, and Marie-Paule Lefranc. 2005. “IMGT/GENE-DB: A Comprehensive Database for Human and Mouse Immunoglobulin and T Cell Receptor Genes.” *Nucleic Acids Research* 33 (suppl_1): D256–61. doi:10.1093/nar/gki010.

Luo, Shishi, Jane A. Yu, and Yun S. Song. 2016. “Estimating Copy Number and Allelic Variation at the Immunoglobulin Heavy Chain Locus Using Short Reads.” *PLOS Computational Biology* 12 (9): e1005117. doi:10.1371/journal.pcbi.1005117.

Mallick, Swapan, Heng Li, Mark Lipson, Iain Mathieson, Melissa Gymrek, Fernando Racimo, Mengyao Zhao, Niru Chennagiri, Susanne Nordenfelt, Arti Tandon, et al. 2016. “The Simons Genome Diversity Project: 300 Genomes from 142 Diverse Populations.” *Nature* 538 (7624): 201–6. doi:10.1038/nature18964.

Olivieri, David N, and Francisco Gambón-Deza. 2014. “VgeneRepertoire.org Identifies and Stores Variable Genes of Immunoglobulins and T-Cell Receptors from the Genomes of Jawed Vertebrates.” *bioRxiv*, January. doi:10.1101/002139.
