# Supplementary material for: Worldwide genetic variation of the IGHV and TRBV immune receptor gene families in humans
Source: Life Sci Alliance. 2019 Feb 26;2(2):e201800221. doi: 10.26508/lsa.201800221 (PMC6391684; doi:10.26508/lsa.201800221)
Supplement: Supplementary file 2 [file LSA-2018-00221_Supplementary_Information.zip › Figure_data_1.pdf]

Coverage profile	Notes	Copy number distribution

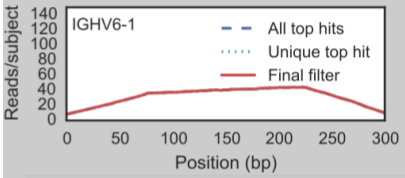

No subsequences shared with other known IGHV genes.

Predominantly single copy per haplotype.

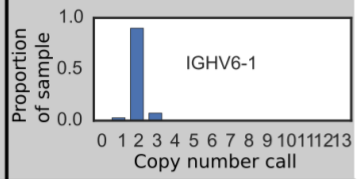

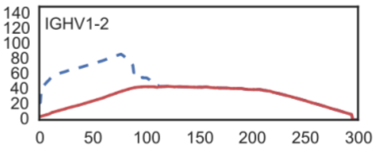

Shares subsequences with: IGHV1-8, IGHV1-OR15-1.

Predominantly single copy per haplotype.

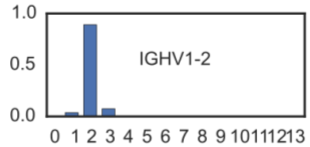

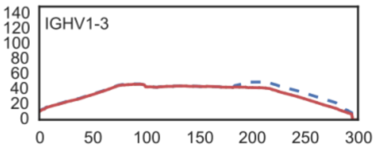

Shares subsequences with: IGHV1-46 (with muts), IGHV1-OR15-2 (with muts).

Predominantly single copy per haplotype.

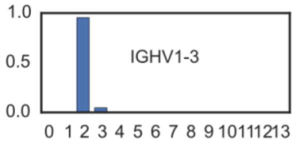

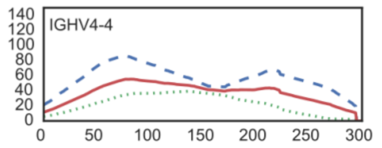

Shares subsequences with: IGHV4-28, IGHV4-30-2, IGHV4-38-2, IGHV4-39, IGHV4-59, IGHV4-61, IGHV4-OR15-8.

High similarity with other functional genes and uneven read coverage profile makes calling copy number problematic.

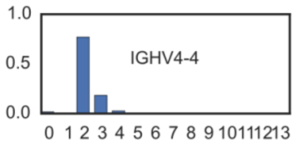

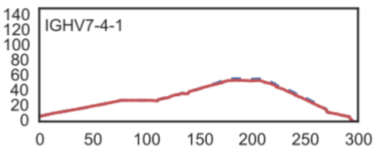

No subsequences shared with other known IGHV genes, but inflated read coverage in 125-250 bp region.

Present in 0 or 1 copy per haplotype (after adjusting for inflated coverage).

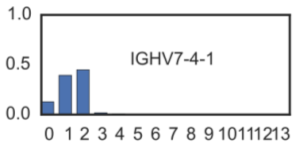

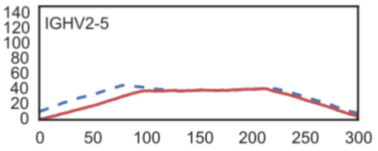

Shares subsequences with: IGHV2-20, IGHV2-70D.

Predominantly single copy per haplotype.

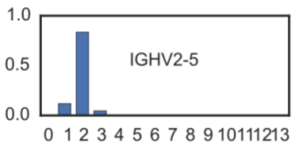

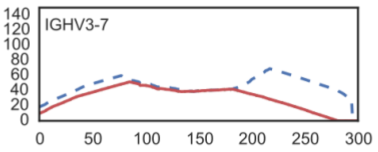

Shares subsequences with: IGHV3-11, IGHV3-21, IGHV3-23, IGHV23D (with muts), IGHV3-48, IGHV3-64, IGHV3-66, IGHV3-69-1(P), IGHV3-71(P).

Predominantly single copy per haplotype.

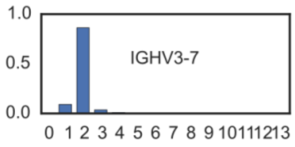

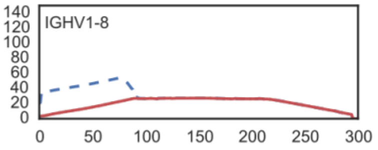

Shares subsequences with: IGHV1-2, IGHV1-OR15-1.

Present in 0 or 1 copy per haplotype.

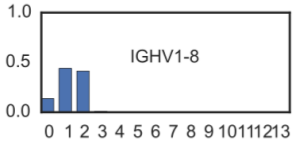

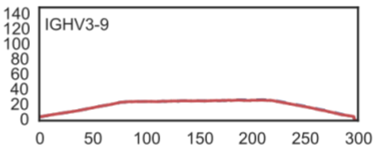

No subsequences shared with other known IGHV genes.

Present in 0 or 1 copy per haplotype.

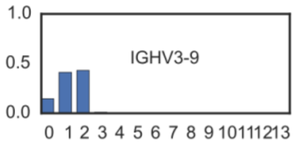

Supplementary Information Figure 1 (part 1 of 5). Detailed characteristics of each operationally distinguishable IGHV gene segment. Each row corresponds to a segment. The first column shows the per-base read coverage of the segment, averaged over 109 subjects (from blood/saliva samples). Blue dashed line is the coverage if we count all reads for which the segment is a top hit (an over estimate of actual coverage). Green dotted line is the coverage if we only count reads for which the segment is the unique top hit (an underestimate of coverage). Red solid line is the coverage after we apply the appropriate filter, customized for that segment (details in the Supplementary Text, Read filtering). A segment which does not share subsequences with any other segment should display a trapezoidal shape. The second column notes any operationally indistinguishable segments, whether there are any segments for which subsequences are shared (this is defined to be at least 75 bp of identical subsequence), and what the typical copy number of the segment is. If there are segments with which subsequences with a few mutations are shared, these are listed with "with muts" in parentheses. The third column displays the distribution of copy number for that segment. The two-copy segments that are used for allele and single nucleotide calls are shaded in grey.

Coverage profile	Notes	Copy number distribution
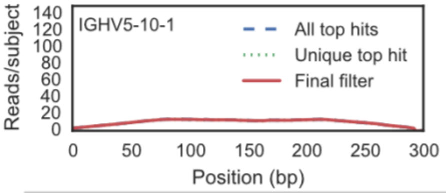	No subsequences shared with other known IGHV genes.<br><br>Present in 0 or 1 copy per haplotype.	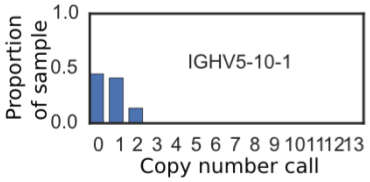
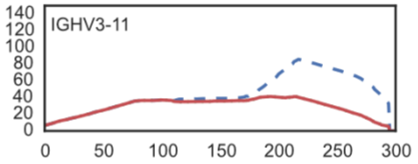	Shares subsequences with: IGHV3-7, IGHV3-21, IGHV3-48, IGHV3-69-1(P).<br><br>Predominantly single copy per haplotype.	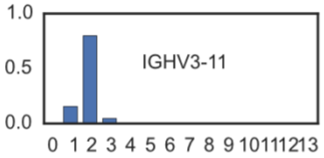
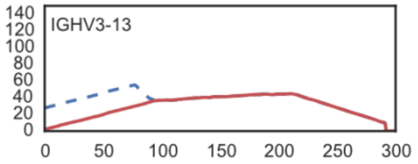	Shares subsequences with: IGHV3-16(ORF), IGHV3-22(P), IGHV3-23, IGHV3-48.<br><br>Predominantly single copy per haplotype.	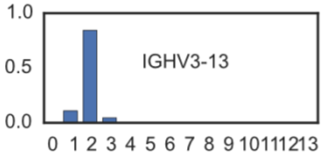
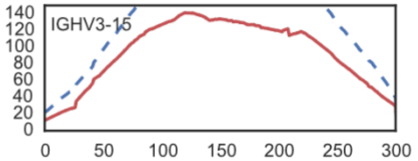	Shares subsequence with IGHV3-OR16-1. However, even after adjusting for this, we still observe an over-abundance of reads (see coverage profile on left).<br><br>If we use GRCh37-mapped reads instead, we observe predominantly single copy per haplotype (histogram on right).	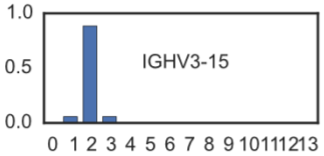
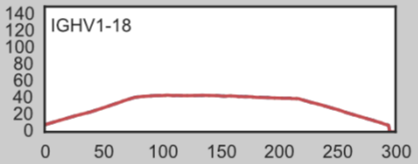	No subsequences shared with other known IGHV genes.<br><br>Predominantly single copy per haplotype.	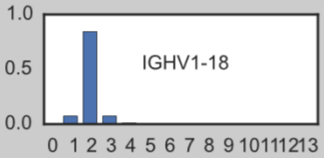
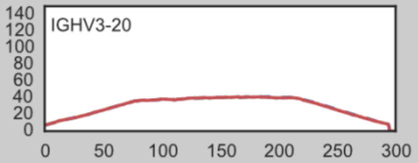	No subsequences shared with other known IGHV genes.<br><br>Predominantly single copy per haplotype.	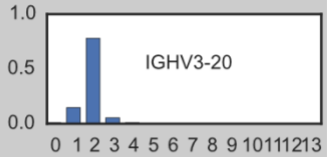
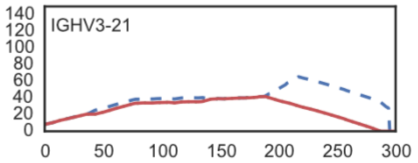	Shares subsequences with: IGHV3-7, IGHV3-11, IGHV3-48, IGHV3-69-1(P).<br><br>Predominantly single copy per haplotype.	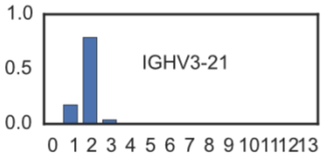
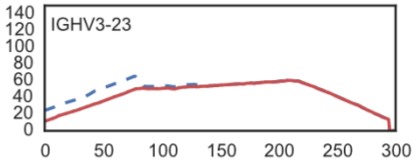	Operationally indistinguishable from IGHV3-23D.<br><br>Shares subsequences with: IGHV3-7 (with muts), IGHV3-13, IGHV3-16(ORF), IGHV3-22(P), IGHV3-23D, IGHV3-48.<br><br>Ranges from 1 to 7 per individual (approx 1-4 per haplotype).	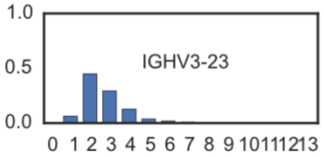
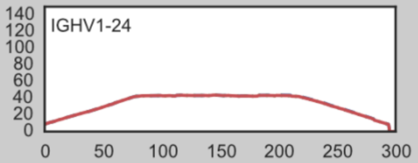	No subsequences shared with other known IGHV genes.<br><br>Predominantly single copy per haplotype.	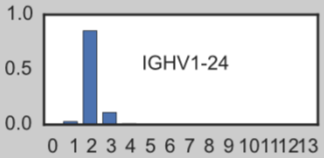

Supplementary Information Figure 1 (part 2 of 5)

Coverage profile	Notes	Copy number distribution
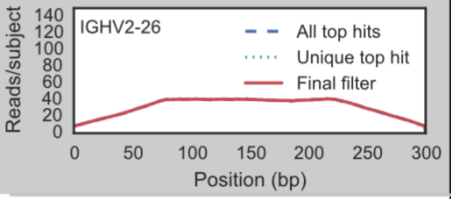	No subsequences shared with other known IGHV genes.<br><br>Predominantly single copy per haplotype.	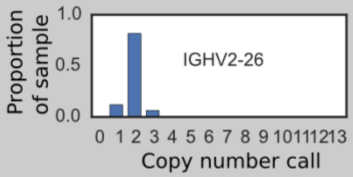
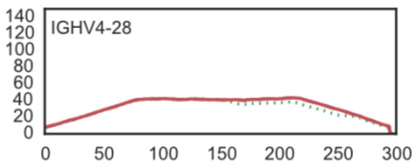	Shares subsequence with IGHV4-4, IGHV4-55(P), IGHV4-59.<br><br>Predominantly single copy per haplotype.	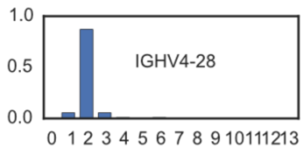
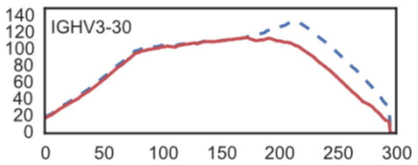	Operationally indistinguishable from IGHV3-30-3, IGHV3-30-5, IGHV3-33.<br><br>Shares subsequence with IGHV3-66, IGHV3-NL1.<br><br>Ranges from 1-13 per individual (approx 1-7 per haplotype).	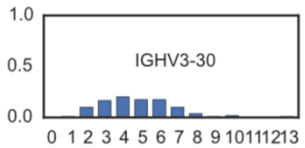
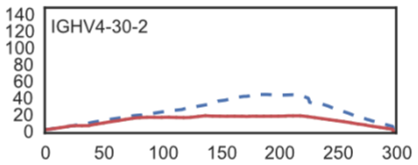	Shares subsequence with IGHV4-4, IGHV4-30-4, IGHV4-38-2, IGHV4-39, IGHV4-59, IGHV4-61.<br><br>High similarity with other functional genes makes calling copy number problematic.	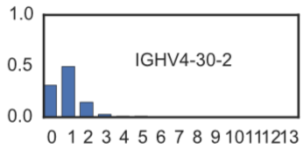
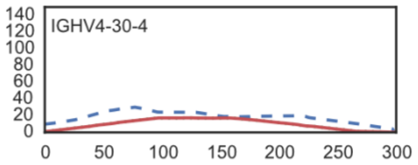	Shares subsequence with IGHV4-30-2, IGHV4-31, IGHV4-59, IGHV4-61.<br><br>High similarity with other functional genes makes calling copy number problematic.	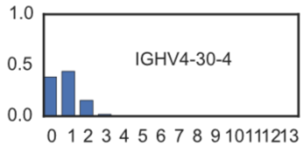
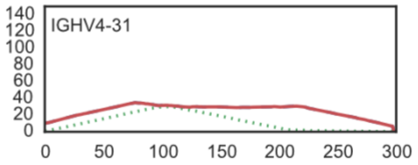	Shares subsequence with: IGHV4-30-4, IGHV4-34, IGHV4-59, IGHV4-61.<br><br>High similarity with other functional genes makes calling copy number problematic.	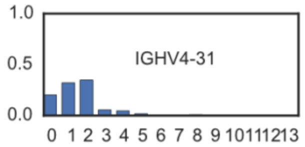
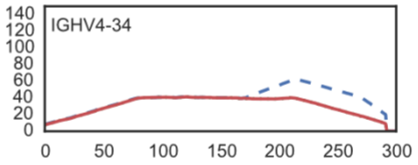	Shares subsequence with IGHV4-31, IGHV4-39, IGHV4-55(P), IGHV4-59, IGHV4-61.<br><br>Predominantly single copy per haplotype.	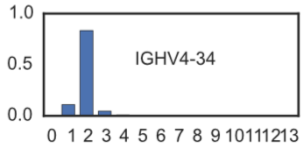
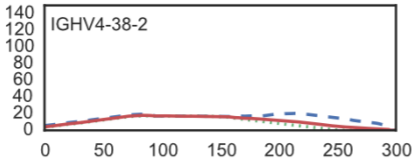	Shares subsequence with IGHV4-4, IGHV4-30-2, IGHV4-39, IGHV4-55(P) (with muts), IGHV4-59, IGHV4-61.<br><br>Present in 0 or 1 copy per haplotype.	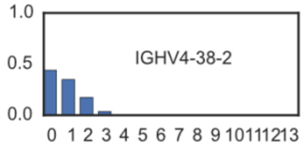
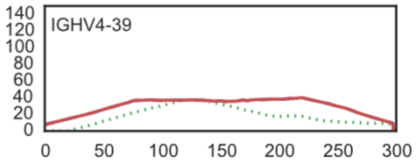	Shares subsequence with IGHV4-4, IGHV4-30-2, IGHV4-34, IGHV4-38-2, IGHV4-59, IGHV4-61.<br><br>Predominantly single copy per haplotype.	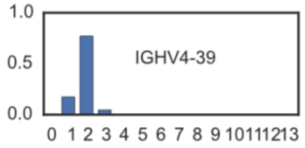

Supplementary Information Figure 1 (part 3 of 5)

Coverage profile | **Notes** | **Copy number distribution**

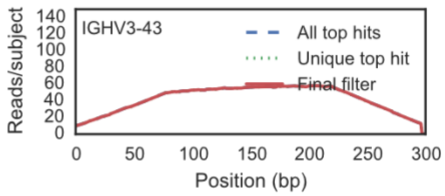

Operationally indistinguishable from IGHV3-43D.

No subsequences shared with other known IGHV genes.

Ranges from 2-6 per individual (approx 1-3 per haplotype).

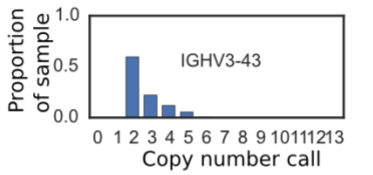

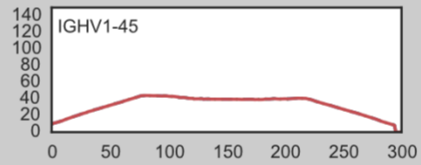

No subsequences shared with other known IGHV genes.

Predominantly single copy per haplotype.

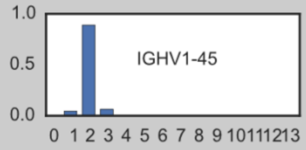

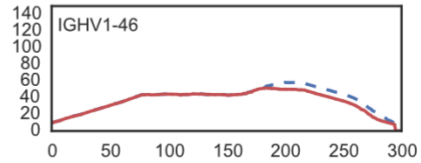

Shares subsequences with: IGHV1-3 (with muts), IGHV1-OR15-2 (with muts)

Predominantly single copy per haplotype.

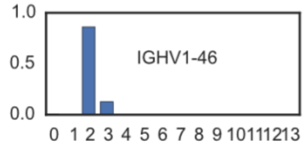

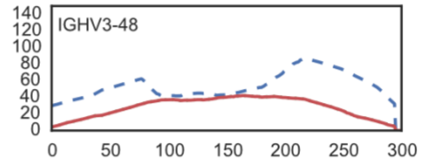

Shares subsequences with: IGHV3-7, IGHV3-11, IGHV3-13, IGHV3-16, IGHV3-21, IGHV3-22(P), IGHV3-23, IGHV3-69-1(P)

Predominantly single copy per haplotype.

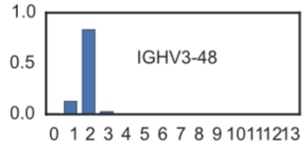

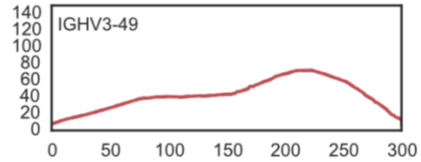

No subsequences shared with other known IGHV genes, but inflated read coverage in 175-275 bp region.

Predominantly single copy per haplotype (after adjusting for inflated coverage).

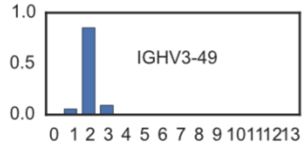

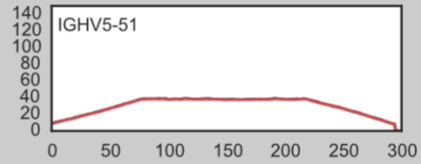

No subsequences shared with other known IGHV genes.

Predominantly single copy per haplotype.

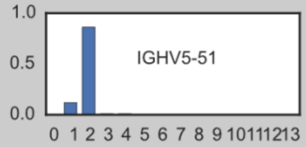

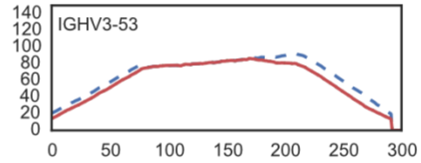

Operationally indistinguishable from IGHV3-66.

Shares subsequences with: IGHV3-7, IGHV3-30, IGHV3-64, IGHV3-71.

Though the histogram on the right suggests 3-4 copies per individual, we see only one main cluster in scaled read coverage data, so we believe it is actually predominantly 2 copies per haplotype.

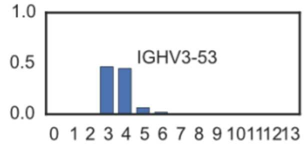

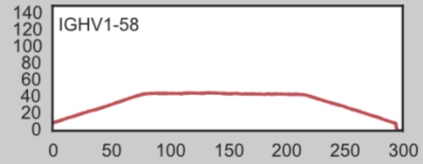

No subsequences shared with other known IGHV genes.

Predominantly single copy per haplotype.

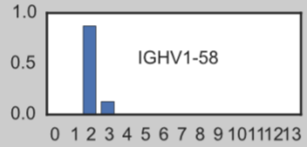

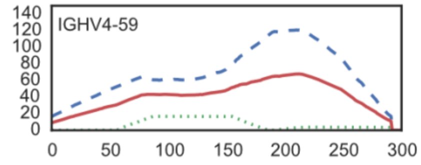

Shares subsequence with: IGHV4-4, IGHV4-28, IGHV4-30-2, IGHV4-31, IGHV4-34, IGHV4-38-2, IGHV4-39, IGHV4-61.

High similarity with other functional genes makes calling copy number problematic.

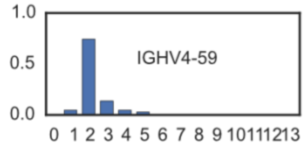

Supplementary Information Figure 1 (part 4 of 5)

Coverage profile	Notes	Copy number distribution
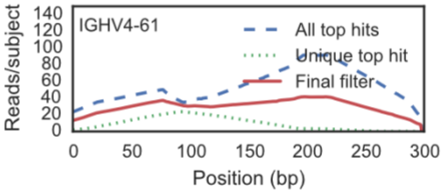	Shares subsequence with: IGHV4-4, IGHV4-30-2, IGHV4-30-4, IGHV4-31, IGHV4-34, IGHV4-38-2, IGHV4-39, IGHV4-59, IGHV4-OR15-8.<br><br>High similarity with other functional genes makes calling copy number problematic.	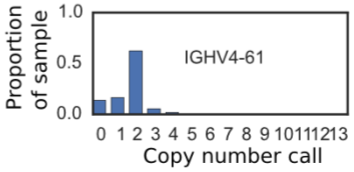
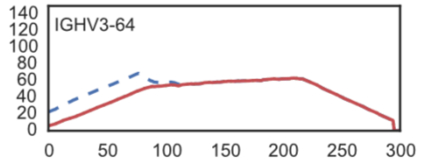	Operationally indistinguishable from IGHV3-64D.<br><br>Shares subsequences with: IGHV3-7, IGHV3-62(P), IGHV3-66, IGHV3-71, IGHV3-OR16-10 (with muts)<br><br>Ranges from 2-4 per individual (approx 1-2 per haplotype).	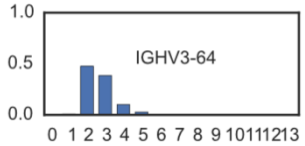
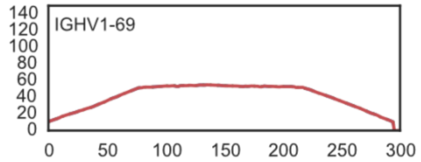	Operationally indistinguishable from IGHV1-69D.<br><br>No subsequences shared with other known IGHV genes.<br><br>Ranges from 2-4 per individual (approx 1-2 per haplotype).	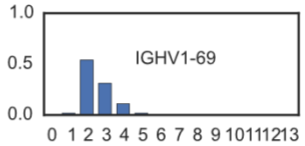
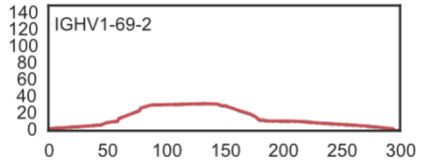	No subsequences shared with other known IGHV genes, but inflated read coverage in 75-175 bp region.<br><br>Present in 0 or 1 copy per haplotype (after adjusting for inflated coverage).	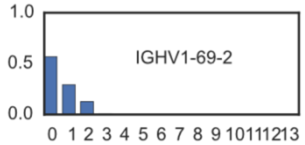
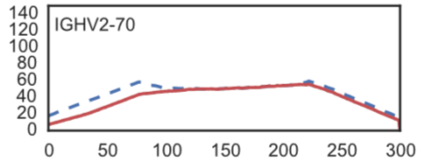	Operationally indistinguishable from IGHV2-70D.<br><br>Shares subsequences with: IGHV2-5.<br><br>Ranges from 2-4 per individual (approx 1-2 per haplotype).	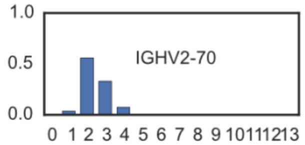
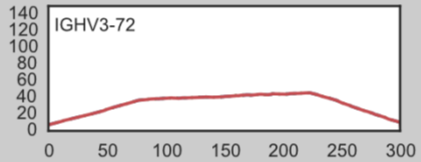	No subsequences shared with other known IGHV genes.<br><br>Predominantly single copy per haplotype.	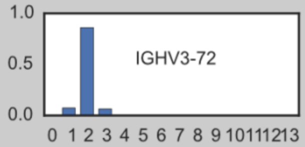
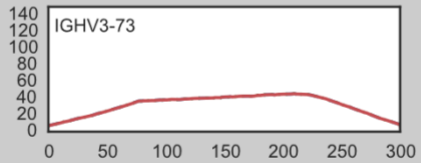	No subsequences shared with other known IGHV genes.<br><br>Predominantly single copy per haplotype.	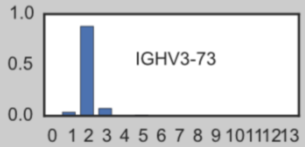
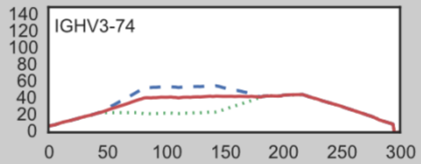	Shares subsequence with: IGHV3-OR16-13.<br><br>Predominantly single copy per haplotype.	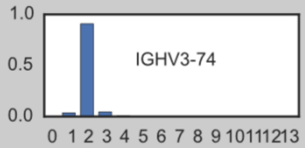
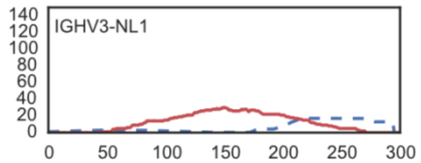	Shares subsequences with: IGHV3-30, IGHV3-30-3.<br><br>Final filter in coverage profile (left, red solid line) is for the sole individual for which IGHV3-NL1 is present (as one copy on one haplotype).	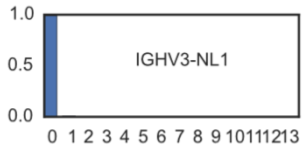

Supplementary Information Figure 1 (part 5 of 5)
