# Supplementary material for: Worldwide genetic variation of the IGHV and TRBV immune receptor gene families in humans
Source: Life Sci Alliance. 2019 Feb 26;2(2):e201800221. doi: 10.26508/lsa.201800221 (PMC6391684; doi:10.26508/lsa.201800221)
Supplement: Supplementary file 2 [file LSA-2018-00221_Supplementary_Information.zip › Figure_data_2.pdf]

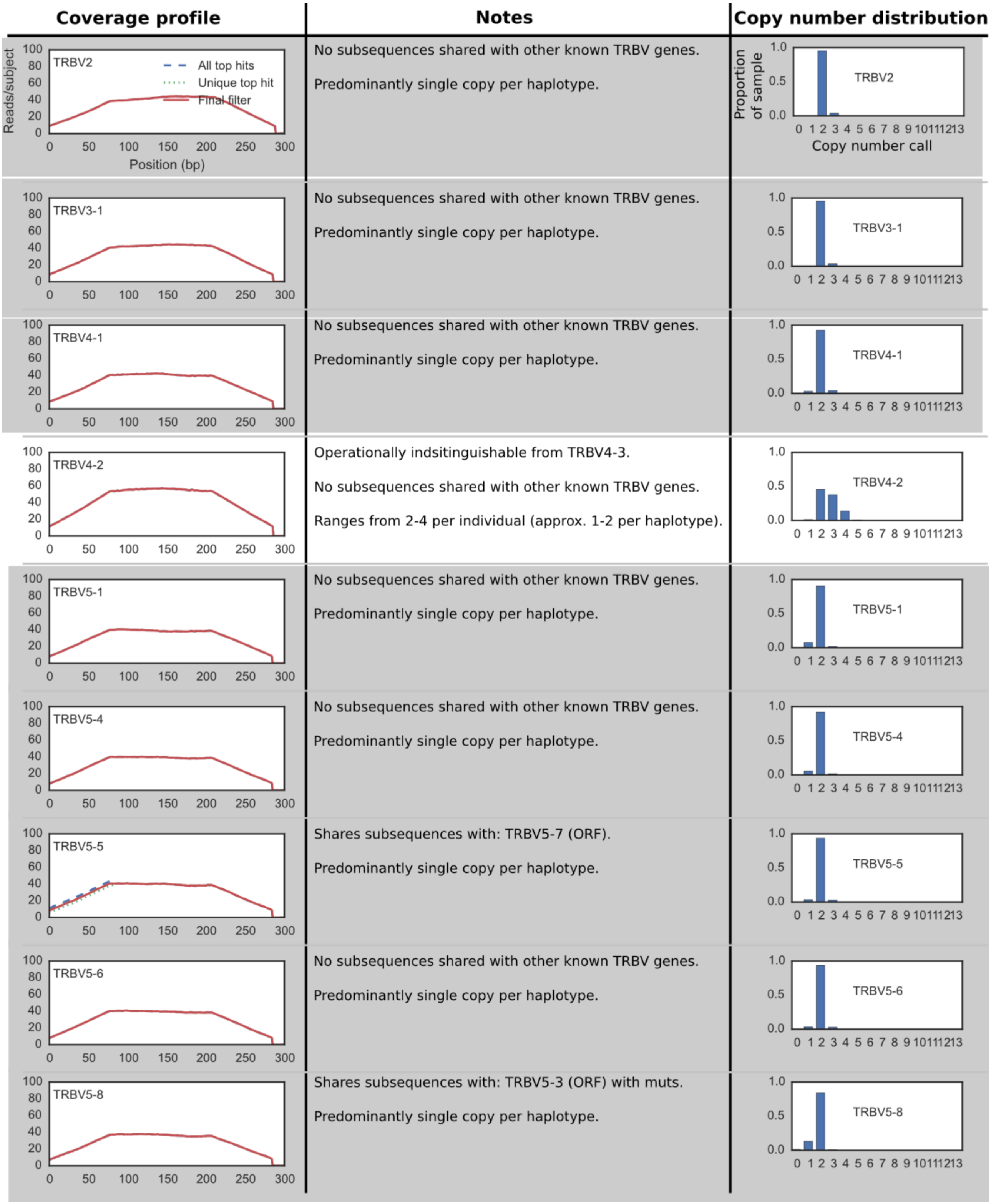

Supplementary Information Figure 2 (part 1 of 5). Detailed characteristics of each operationally distinguishable TRBV gene segment. Each row corresponds to a segment. The first column shows the per-base read coverage of the segment, averaged over 286 subjects. Blue dashed line is the coverage if we count all reads for which the segment is a top hit (an over estimate of actual coverage). Green dotted line is the coverage if we only count reads for which the segment is the unique top hit (an underestimate of coverage). Red solid line is the coverage after we apply the appropriate filter, customized for that segment (details in Supplementary Text). A segment which does not share subsequences with any other segment should display a trapezoidal shape. The second column notes any operationally indistinguishable segments, whether there are any segments for which subsequences are shared (this is defined to be at least 75 bp of identical subsequence), and what the typical copy number of the segment is. If there are segments with which subsequences with a few mutations are shared, these are listed with “with muts” in parentheses. The third column displays the distribution of copy number for that segment. The two-copy segments that are used for allele and single nucleotide calls are shaded in grey.

Coverage profile	Notes	Copy number distribution
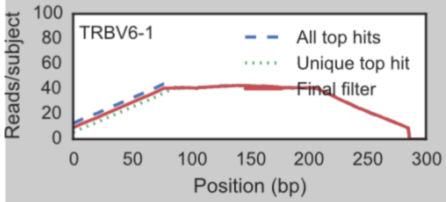	Shares subsequences with: TRBV6-5.<br><br>Predominantly single copy per haplotype.	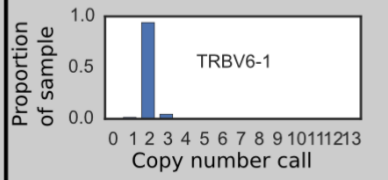
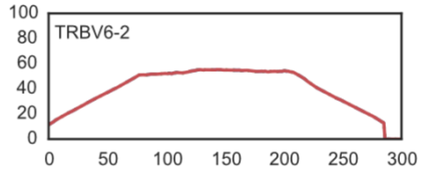	Operationally indistinguishable from TRBV6-3.<br><br>No subsequences shared with other known TRBV genes.<br><br>Ranges from 2-4 per individual (approx. 1-2 per haplotype.	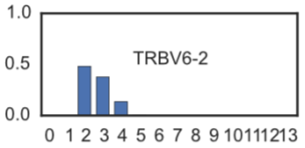
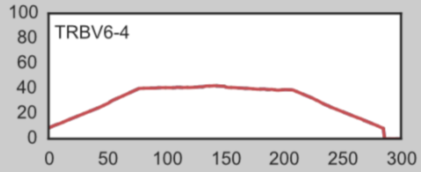	No subsequences shared with other known TRBV genes.<br><br>Predominantly single copy per haplotype.	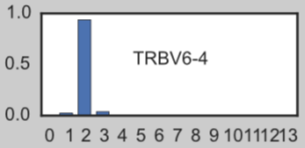
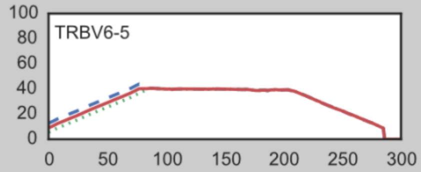	Shares subsequences with: TRBV6-1.<br><br>Predominantly single copy per haplotype.	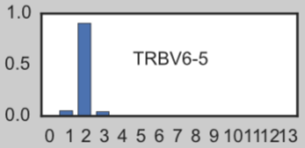
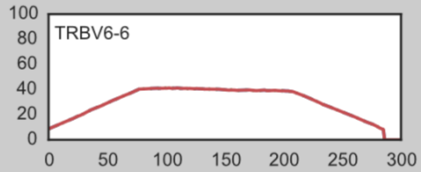	No subsequences shared with other known TRBV genes.<br><br>Predominantly single copy per haplotype.	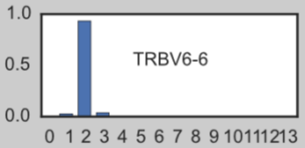
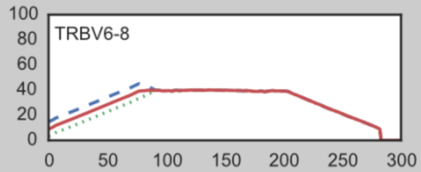	Shares subsequence with: TRBV6-9.<br><br>Predominantly single copy per haplotype.	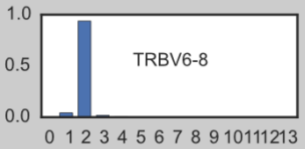
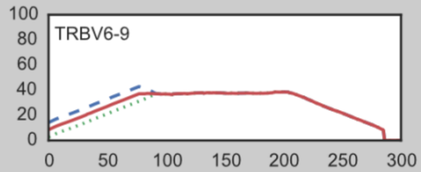	Shares subsequence with: TRBV6-8.<br><br>Predominantly single copy per haplotype.	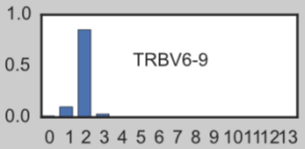
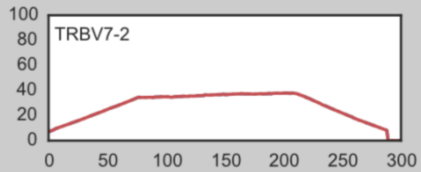	No subsequences shared with other known TRBV genes.<br><br>Predominantly single copy per haplotype.	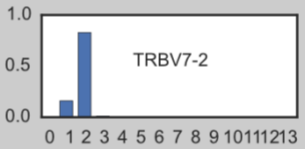
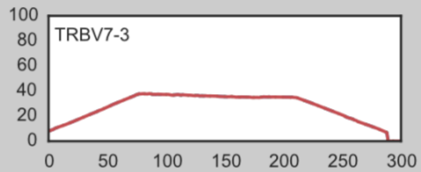	No subsequences shared with other known TRBV genes.<br><br>Predominantly single copy per haplotype.	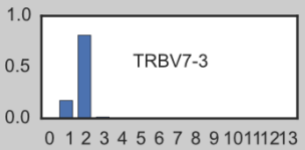

Supplementary Information Figure 2 (part 2 of 5).

Coverage profile	Notes	Copy number distribution
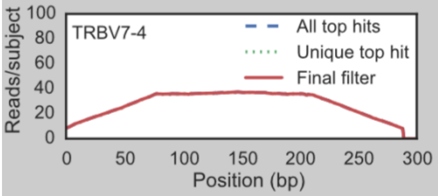	Shares subsequences with; TRBV7-6 (with muts).<br><br>Predominantly single copy per haplotype.	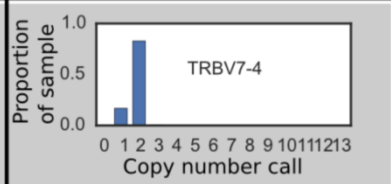
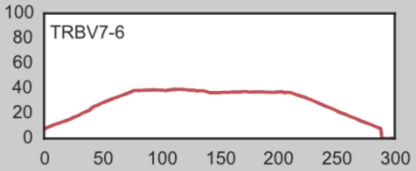	Shares subsequences with; TRBV7-4 (with muts).<br><br>Predominantly single copy per haplotype.	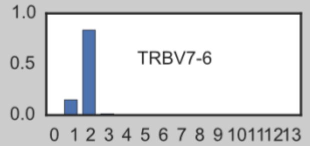
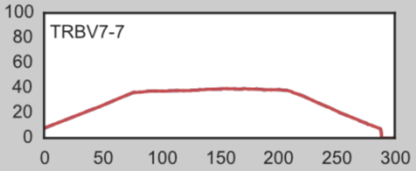	No subsequences shared with other known TRBV genes.<br><br>Predominantly single copy per haplotype.	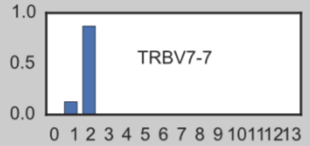
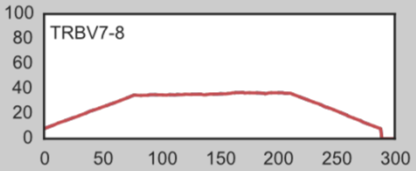	No subsequences shared with other known TRBV genes.<br><br>Predominantly single copy per haplotype.	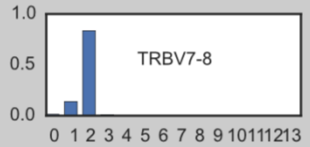
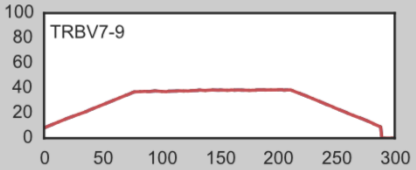	No subsequences shared with other known TRBV genes.<br><br>Predominantly single copy per haplotype.	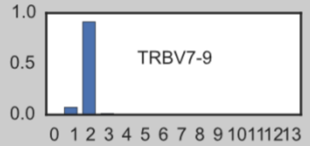
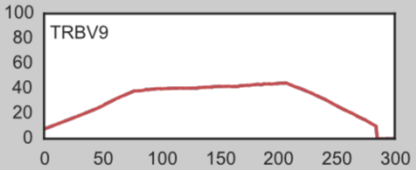	No subsequences shared with other known TRBV genes.<br><br>Predominantly single copy per haplotype.	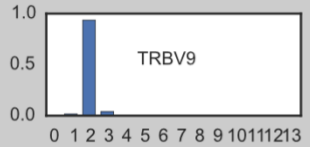
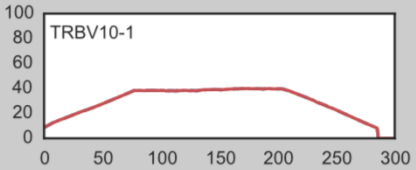	No subsequences shared with other known TRBV genes.<br><br>Predominantly single copy per haplotype.	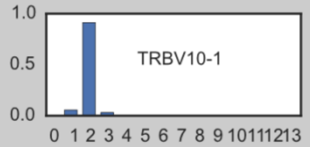
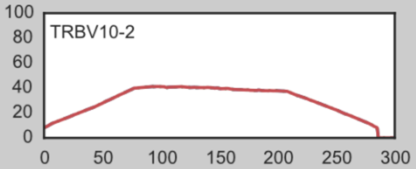	No subsequences shared with other known TRBV genes.<br><br>Predominantly single copy per haplotype.	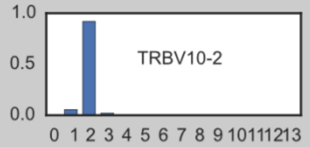
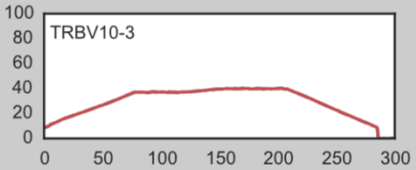	No subsequences shared with other known TRBV genes.<br><br>Predominantly single copy per haplotype.	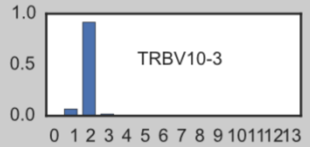

Supplementary Information Figure 2 (part 3 of 5).

Coverage profile	Notes	Copy number distribution
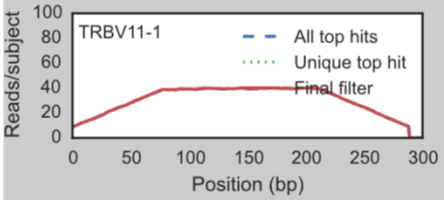	No subsequences shared with other known TRBV genes.<br>Predominantly single copy per haplotype.	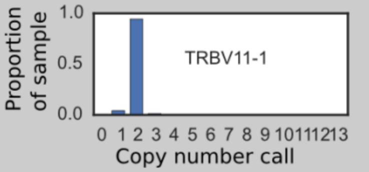
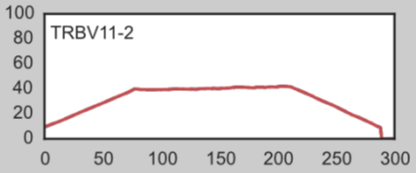	No subsequences shared with other known TRBV genes.<br>Predominantly single copy per haplotype.	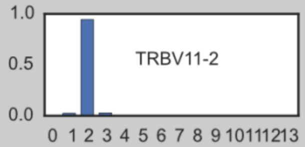
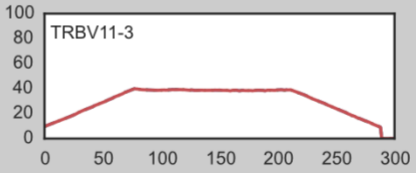	No subsequences shared with other known TRBV genes.<br>Predominantly single copy per haplotype.	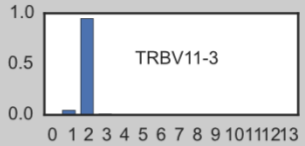
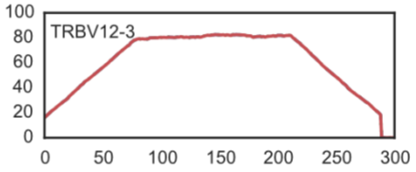	Operationally indistinguishable from TRBV12-4.<br>No subsequences shared with other known TRBV genes.<br>Predominantly two copies per haplotype.	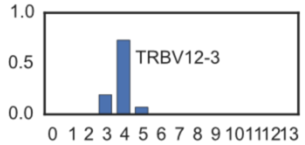
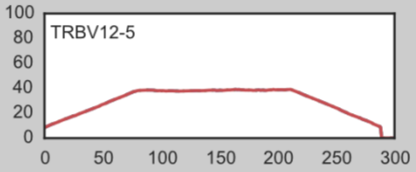	No subsequences shared with other known TRBV genes.<br>Predominantly single copy per haplotype.	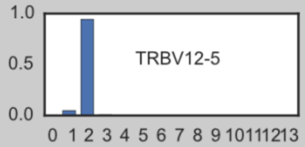
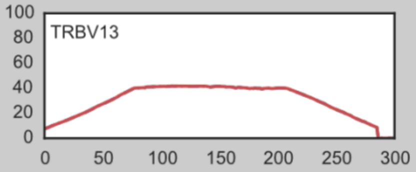	No subsequences shared with other known TRBV genes.<br>Predominantly single copy per haplotype.	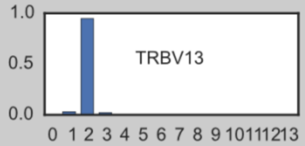
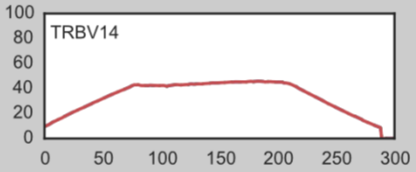	No subsequences shared with other known TRBV genes.<br>Predominantly single copy per haplotype.	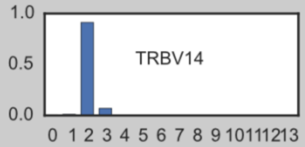
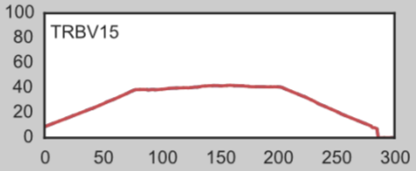	No subsequences shared with other known TRBV genes.<br>Predominantly single copy per haplotype.	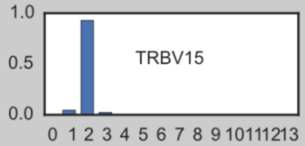
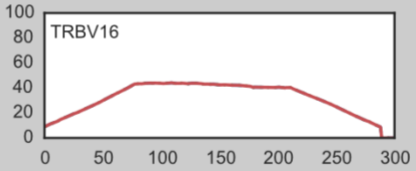	No subsequences shared with other known TRBV genes.<br>Predominantly single copy per haplotype.	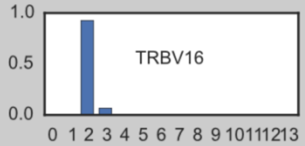

Supplementary Information Figure 2 (part 4 of 5).

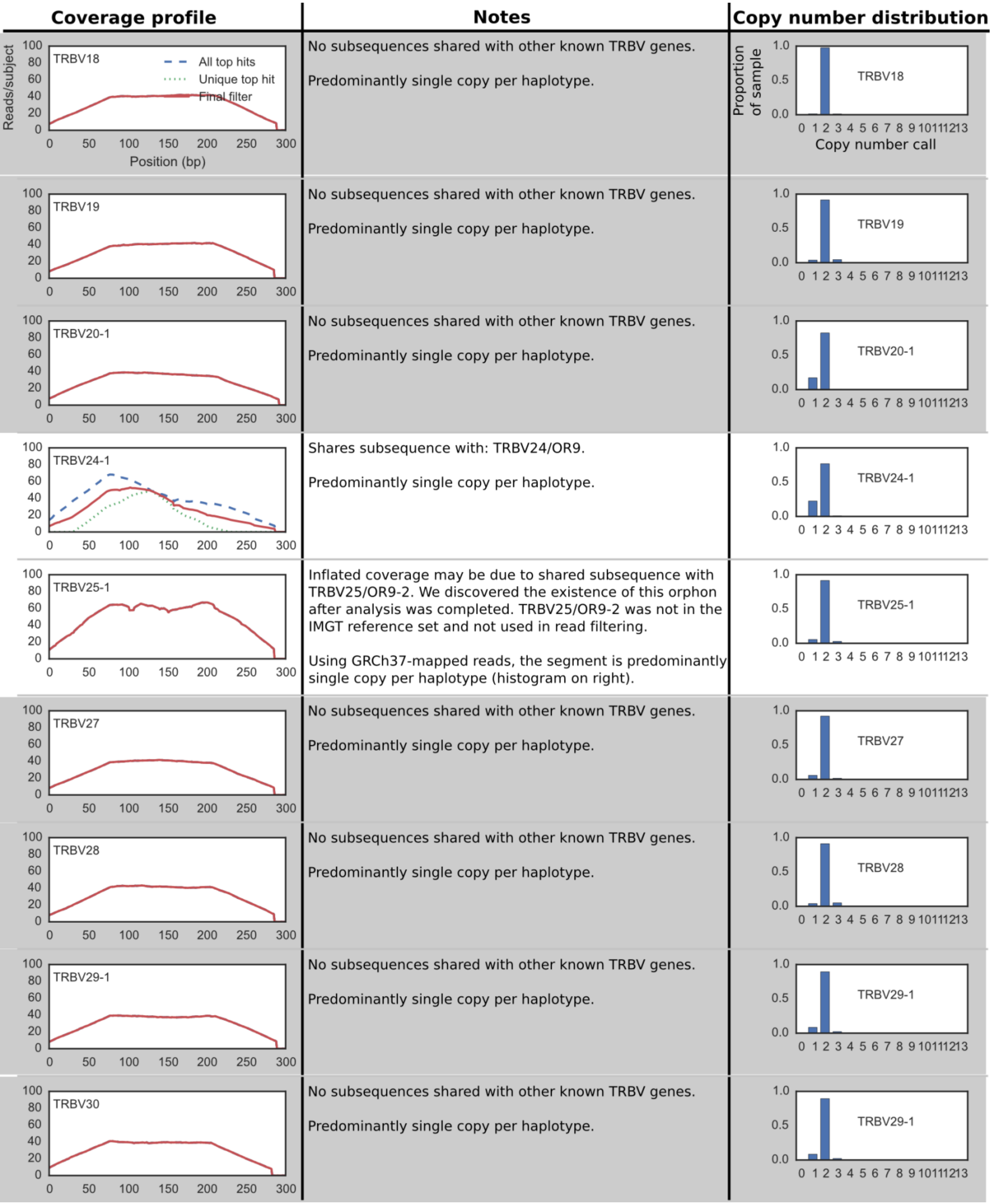

Supplementary Information Figure 2 (part 5 of 5).
