# Supplementary material for: Worldwide genetic variation of the IGHV and TRBV immune receptor gene families in humans
Source: Life Sci Alliance. 2019 Feb 26;2(2):e201800221. doi: 10.26508/lsa.201800221 (PMC6391684; doi:10.26508/lsa.201800221)
Supplement: Supplementary file 2 [file LSA-2018-00221_Supplementary_Information.zip › Figure_data_4.pdf]

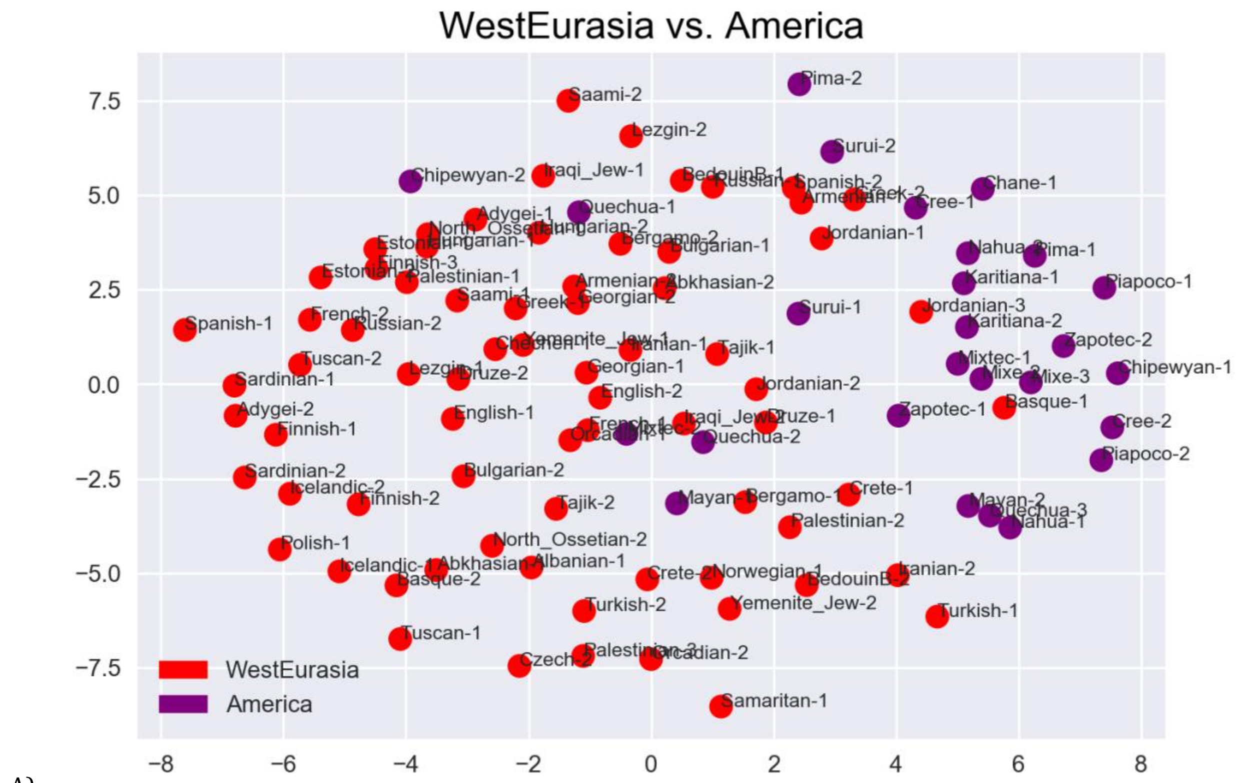

A)

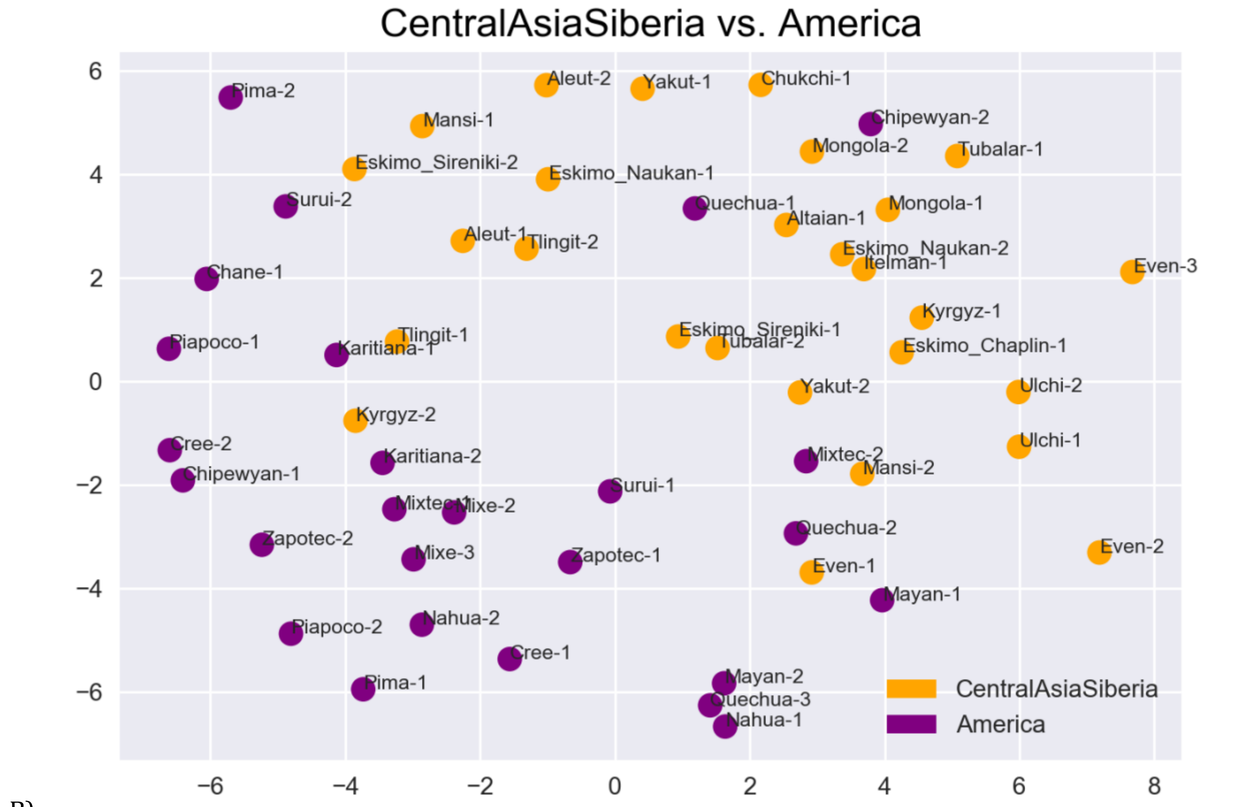

B)

Supplementary Information Figure 4 (part 1 of 3). Multidimensional scaling of 26 Native Americans and A) 73 West Eurasians, B) 27 Central Asians or Siberians, C) 45 East Asians, D) 49 South Asians, and E) 22 Oceanians. For comparison of Native Americans and Africans, refer to panel F) of Fig. S9. Data used includes all DNA source types in the SGDP dataset. The metric used for scaling was based on the Euclidean distance between the set of alleles of each individual. Individuals are labeled by an abbreviated version of their sample ID in the SGDP dataset.

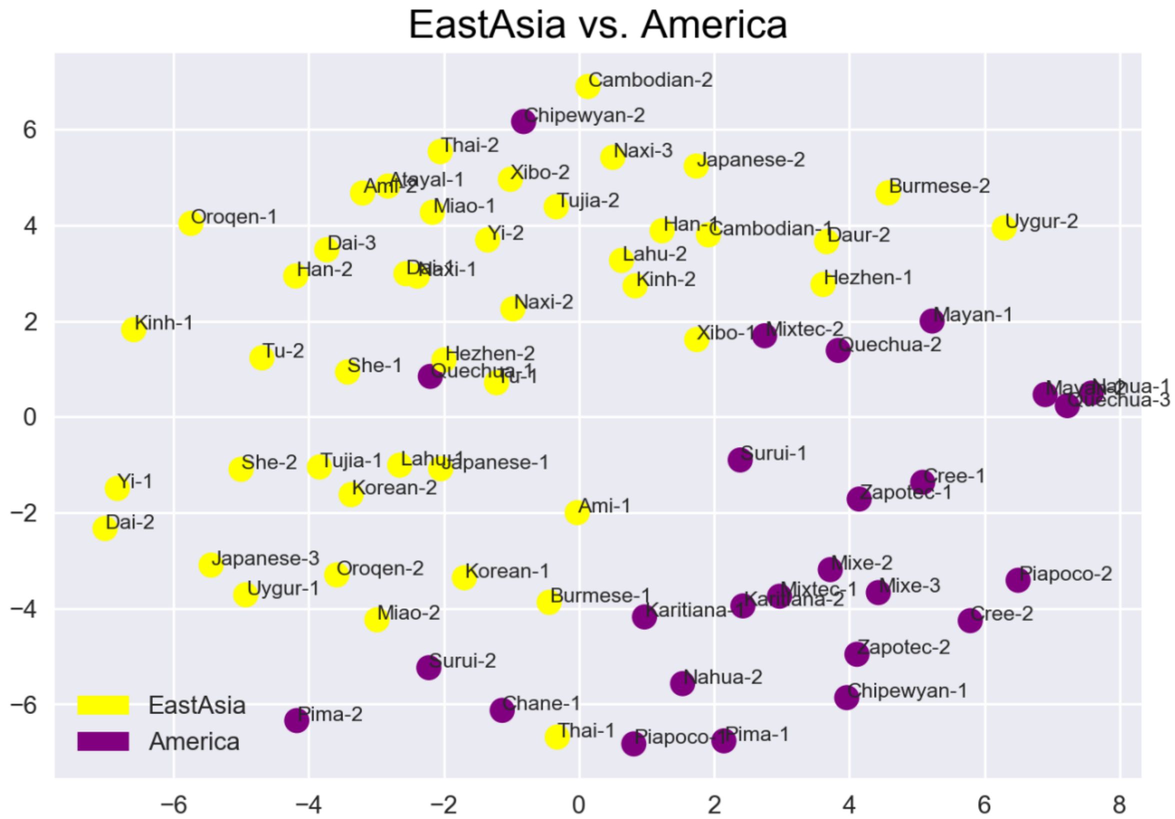

C)

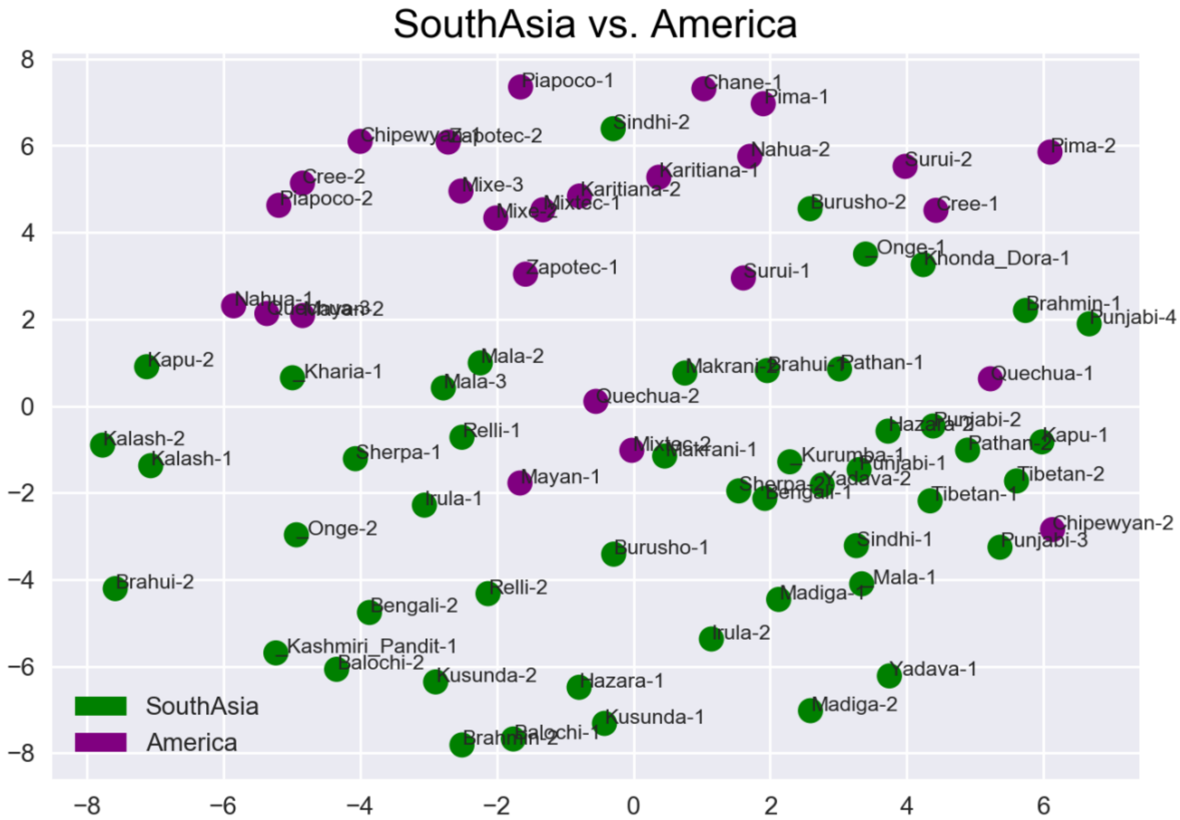

D)

Supplementary Information Figure 4 (part 2 of 3).

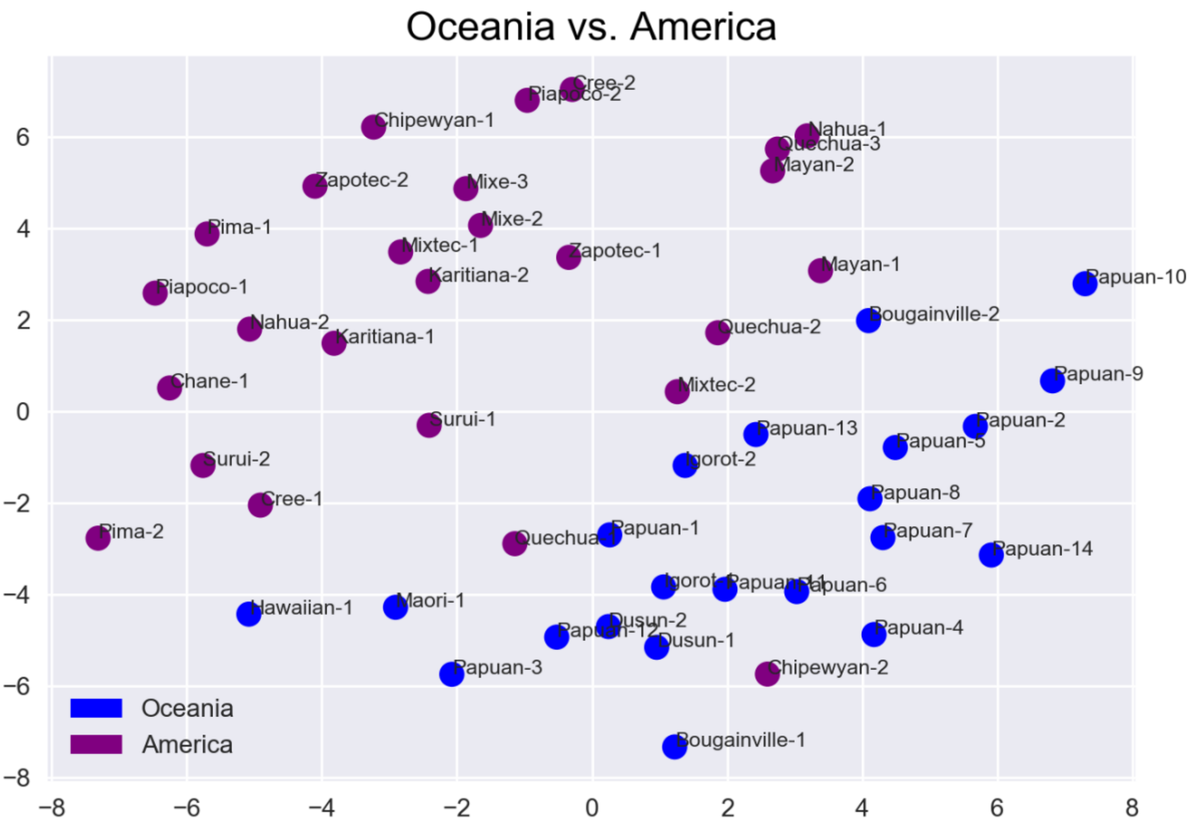

E)

Supplementary Information Figure 4 (part 3 of 3).
