# Supplementary material for: Worldwide genetic variation of the IGHV and TRBV immune receptor gene families in humans
Source: Life Sci Alliance. 2019 Feb 26;2(2):e201800221. doi: 10.26508/lsa.201800221 (PMC6391684; doi:10.26508/lsa.201800221)
Supplement: Supplementary file 3 [file LSA-2018-00221_TableS1.pdf]

Allele name	Allele frequency
IGHV1-18*01	0.824
IGHV1-18*04	0.167
IGHV1-18*01_ag168ND	0.009
IGHV1-24*01	1.0
IGHV1-45*02	0.839
IGHV1-45*02_ga123GR	0.161
IGHV1-58*01	0.465
IGHV1-58*02	0.498
IGHV1-58*02_gt57VF	0.037
IGHV2-26*01	0.821
IGHV2-26*01_ct257NN_ct294RW	0.113
IGHV2-26*01_ct257NN	0.066
IGHV3-20*01	0.561
IGHV3-20*01_ct282HY	0.198
IGHV3-20*01_gt64CF	0.231
IGHV3-20*01_ag88DG_ct282HY	0.009
IGHV3-72*01	0.991
IGHV3-72*01_tc170SS	0.009
IGHV3-73*01	0.502
IGHV3-73*02	0.479
IGHV3-73*01_ag55KR	0.018
IGHV3-74*01	0.986
IGHV3-74*02	0.014
IGHV5-51*01	0.824
IGHV5-51*03	0.157
IGHV5-51*01_ga112RH	0.019
IGHV6-1*01	0.963
IGHV6-1*01_ct207R_ (P)	0.037
