# Supplementary material for: Worldwide genetic variation of the IGHV and TRBV immune receptor gene families in humans
Source: Life Sci Alliance. 2019 Feb 26;2(2):e201800221. doi: 10.26508/lsa.201800221 (PMC6391684; doi:10.26508/lsa.201800221)
Supplement: Supplementary file 4 [file LSA-2018-00221_TableS2.pdf]

Allele name	Freq.
TRBV10-1*01	0.634
TRBV10-1*02_ gt234E_ (P)	0.239
TRBV10-1*02	0.127
TRBV10-2*01	0.894
TRBV10-2*01_tc191YY (T)	0.106
TRBV10-3*01	0.39
TRBV10-3*02	0.462
TRBV10-3*03	0.124
TRBV10-3*01_ ga118GE (T)	0.024
TRBV11-1*01	0.94
TRBV11-1*01_ ag85HR_ct98YY_ag142QR	0.06
TRBV11-2*01	0.662
TRBV11-2*03	0.338
TRBV11-3*01	0.885
TRBV11-3*02	0.115
TRBV12-5*01	0.551
TRBV12-5*01_ cg27HD	0.417
TRBV12-5*01_ ga154RQ	0.032
TRBV13*01	0.977
TRBV13*01_ ct78PS (T)	0.023
TRBV14*01	0.866
TRBV14*02	0.134
TRBV15*02	0.977
TRBV15*01	0.023
TRBV16*01	0.976
TRBV16*02	0.024
TRBV18*01	0.953
TRBV18*01_ ag75MV	0.047
TRBV19*01	0.875
TRBV19*01_ ag23PP (T)	0.125
TRBV2*01	1.0
TRBV20-1*01	0.444
TRBV20-1*02	0.422
TRBV20-1*05	0.125
TRBV20-1*02_ ga227LL	0.009
TRBV27*01	1.0
TRBV28*01	1.0
TRBV29-1*01	0.944
TRBV29-1*01_ ac246ML (T)	0.056
TRBV3-1*01	1.0
TRBV30*01	0.707
TRBV30*02	0.211
TRBV30*01_ct108R__ct204PS (T)	0.02
TRBV30*02_ ct108R__ct204PS (P,T)	0.02
TRBV30*01_ga33VM (T)	0.014
TRBV30*04	0.027
TRBV4-1*01	0.991
TRBV4-1*01_ cg181PR	0.009
TRBV5-1*01	1.0
TRBV5-4*01	0.991
TRBV5-4*01_tg213YD (T)	0.009
TRBV5-5*02	0.67
TRBV5-5*01	0.33
TRBV5-6*01	0.948
TRBV5-6*01_ta205FY_ct236NN	0.019
TRBV5-6*01_ta205FY	0.014
TRBV5-6*01_tg244LW	0.009
TRBV5-6*01_gt118GV	0.009
TRBV5-8*01_ct236NN (T)	0.09
TRBV5-8*01_ct55AV (T)	0.142
TRBV5-8*01	0.711
TRBV5-8*01_ct55AV_ct236NN (T)	0.057
TRBV6-1*01	0.991
TRBV6-1*01_ag183ND	0.009
TRBV6-4*01	0.869
TRBV6-4*02	0.085
TRBV6-4*02_ga49RQ	0.019
TRBV6-4*02_gc49RP	0.019
TRBV6-4*02_ac51SR	0.009
TRBV6-5*01	1.0
TRBV6-6*01	0.64
TRBV6-6*02	0.327
TRBV6-6*03_gt216DY	0.009
TRBV6-6*01_ga31RH (T)	0.014
TRBV6-6*01_ca278SR (T)	0.009
TRBV6-8*01	0.962
TRBV6-8*01_ag250QR	0.038
TRBV6-9*01	0.787
TRBV6-9*01_ag263VV	0.213
TRBV7-2*02	0.584
TRBV7-2*01	0.416
TRBV7-3*01	0.933
TRBV7-3*01_gt255DY (T)	0.067
TRBV7-4*01	0.954
TRBV7-4*01_ga214RK	0.037
TRBV7-4*01_ct240RC	0.009
TRBV7-6*01	1.0
TRBV7-7*01	1.0
TRBV7-8*01	0.967
TRBV7-8*02	0.024
TRBV7-8*01_tc258SP (T)	0.009
TRBV7-9*01	0.226
TRBV7-9*03	0.747
TRBV7-9*03_ga67CY_ct105R__ag111TA (P)	0.028
TRBV9*01	0.894
TRBV9*02	0.106
