# Supplementary material for: Worldwide genetic variation of the IGHV and TRBV immune receptor gene families in humans
Source: Life Sci Alliance. 2019 Feb 26;2(2):e201800221. doi: 10.26508/lsa.201800221 (PMC6391684; doi:10.26508/lsa.201800221)
Supplement: Supplementary file 5 [file LSA-2018-00221_TableS3.pdf]

Allele name	Africans	WE	CAS	EA	SA	Oceanians	NA	Total
IGHV1-45*02_ga123GR	0.04	0.08	0.26	0.5	0.17	0.25	0.25	0.16
IGHV2-26*01_ct257NN_ct294RW	0.04	0.05	0.24	0.0	0.11	0.0	0.19	0.11
IGHV2-26*01_ct257NN	0.35	0.02	0.09	0.0	0.0	0.14	0.0	0.07
IGHV3-20*01_ct282HY	0.52	0.11	0.09	0.0	0.24	0.25	0.19	0.2
IGHV3-20*01_gt64CF	0.11	0.33	0.26	0.0	0.2	0.0	0.25	0.23
TRBV10-1*02_gt234E_ (P)	0.19	0.28	0.24	0.33	0.15	0.62	0.25	0.24
TRBV10-2*01_tc191YY (T)	0.11	0.13	0.02	0.0	0.21	0.0	0.0	0.11
TRBV11-1*01_ag85HR_ct98YY_ag142QR	0.07	0.16	0.0	0.0	0.02	0.0	0.0	0.06
TRBV12-5*01_cg27HD	0.11	0.4	0.57	0.25	0.5	0.88	0.12	0.42
TRBV18*01_ag75MV	0.36	0.02	0.0	0.0	0.0	0.0	0.0	0.05
TRBV19*01_ag23PP (T)	0.04	0.11	0.18	0.25	0.09	0.0	0.33	0.12
TRBV29-1*01_ac246ML (T)	0.0	0.0	0.16	0.25	0.0	0.0	0.25	0.06
TRBV5-8*01_ct236NN (T)	0.0	0.08	0.07	0.25	0.15	0.0	0.17	0.09
TRBV5-8*01_ct55AV (T)	0.39	0.08	0.07	0.25	0.15	0.0	0.17	0.14
TRBV5-8*01_ct55AV_ct236NN (T)	0.07	0.03	0.0	0.0	0.12	0.0	0.17	0.06
TRBV6-9*01_ag263VV	0.5	0.13	0.09	0.25	0.27	0.0	0.33	0.21
TRBV7-3*01_gt255DY (T)	0.0	0.0	0.0	0.25	0.24	0.0	0.0	0.07
