# Supplementary material for: Worldwide genetic variation of the IGHV and TRBV immune receptor gene families in humans
Source: Life Sci Alliance. 2019 Feb 26;2(2):e201800221. doi: 10.26508/lsa.201800221 (PMC6391684; doi:10.26508/lsa.201800221)
Supplement: Supplementary file 6 [file LSA-2018-00221_TableS4.pdf]

Variant name	Variant type	Region	No. of haplotypes	Regional freq	Global freq
IGHV1-18_g168	SNV	Africa	2	0.07	0.01
IGHV1-58_t57	SNV	Africa	8	0.29	0.04
IGHV2-26_a118	SNV	Africa	2	0.07	0.01
IGHV3-72_c170	SNV	Africa	2	0.07	0.01
IGHV3-74_t20	SNV	Africa	3	0.11	0.01
IGHV1-18*01_ag168ND	Allele	Africa	2	0.07	0.01
IGHV1-58*02_gt57VF	Allele	Africa	8	0.29	0.04
IGHV3-72*01_tc170SS	Allele	Africa	2	0.07	0.01
IGHV3-74*02	Allele	Africa	3	0.12	0.01
TRBV13_t78	SNV	CentralAsiaSiberia	5	0.11	0.02
TRBV20-1_a227	SNV	Africa	2	0.08	0.01
TRBV30_a33	SNV	CentralAsiaSiberia	2	0.08	0.01
TRBV4-1_g181	SNV	Africa	2	0.07	0.01
TRBV5-4_g213	SNV	Africa	2	0.08	0.01
TRBV5-6_t118	SNV	SouthAsia	2	0.04	0.01
TRBV5-6_a205	SNV	Africa	8	0.29	0.04
TRBV5-6_t236	SNV	Africa	4	0.14	0.02
TRBV6-1_g183	SNV	Africa	2	0.08	0.01
TRBV6-6_a31	SNV	Africa	3	0.12	0.01
TRBV6-6_t216	SNV	Africa	2	0.08	0.01
TRBV6-6_a278	SNV	SouthAsia	2	0.04	0.01
TRBV6-8_g250	SNV	Africa	8	0.42	0.04
TRBV7-4_t240	SNV	Africa	2	0.07	0.01
TRBV13*01_ct78PS (T)	Allele	CentralAsiaSiberia	5	0.11	0.02
TRBV20-1*02_ga227LL	Allele	Africa	2	0.08	0.01
TRBV30*01_ga33VM (T)	Allele	CentralAsiaSiberia	2	0.08	0.01
TRBV4-1*01_cg181PR	Allele	Africa	2	0.07	0.01
TRBV5-4*01_tg213YD (T)	Allele	Africa	2	0.08	0.01
TRBV5-6*01_ta205FY_ct236NN	Allele	Africa	4	0.15	0.02
TRBV5-6*01_ta205FY	Allele	Africa	3	0.11	0.01
TRBV5-6*01_gt118GV	Allele	SouthAsia	2	0.04	0.01
TRBV6-1*01_ag183ND	Allele	Africa	2	0.08	0.01
TRBV6-6*03_gt216DY	Allele	Africa	2	0.08	0.01
TRBV6-6*01_ga31RH (T)	Allele	Africa	3	0.12	0.01
TRBV6-6*01_ca278SR (T)	Allele	SouthAsia	2	0.04	0.01
TRBV6-8*01_ag250QR	Allele	Africa	8	0.42	0.04
TRBV7-4*01_ct240RC	Allele	Africa	2	0.07	0.01
