# Supplementary material for: Worldwide genetic variation of the IGHV and TRBV immune receptor gene families in humans
Source: Life Sci Alliance. 2019 Feb 26;2(2):e201800221. doi: 10.26508/lsa.201800221 (PMC6391684; doi:10.26508/lsa.201800221)
Supplement: Supplementary file 7 [file LSA-2018-00221_TableS5.pdf]

	Africans	WE	CAS	EA	SA	Oceanians
WE	0.126					
CAS	0.165	0.0453				
EA	0.195	0.0416	0.0181			
SA	0.0899	0.0082	0.0495	0.0635		
Oceanians	0.152	0.0402	0.0384	0.0188	0.0489	
NA	0.150	0.1336	0.129	0.170	0.121	0.202
