# Supplementary material for: Worldwide genetic variation of the IGHV and TRBV immune receptor gene families in humans
Source: Life Sci Alliance. 2019 Feb 26;2(2):e201800221. doi: 10.26508/lsa.201800221 (PMC6391684; doi:10.26508/lsa.201800221)
Supplement: Supplementary file 8 [file LSA-2018-00221_TableS6.pdf]

Region	IGHV (n=109)	TRBV (n=286)
Africans	0.98	0.00
Native Americans	1.00	0.34
Central Asians or Siberians	0.98	0.08
East Asians	1.00	0.00
Oceanians	1.00	0.00
South Asians	0.99	0.00
West Eurasians	0.98	0.10
